# Microbiota contribute to regulation of the gut-testis axis in seasonal spermatogenesis

**DOI:** 10.1093/ismejo/wraf036

**Published:** 2025-02-25

**Authors:** Zifang Wu, Long Li, Shaoxian Chen, Ye Gong, Yuyan Liu, Tianqi Jin, Yang Wang, Jie Tang, Qian Dong, Bangzhu Yang, Fangxia Yang, Wuzi Dong

**Affiliations:** College of Animal Science and Technology, Northwest A&F University, Yangling, Shaanxi 712100, China; College of Animal Science and Technology, Northwest A&F University, Yangling, Shaanxi 712100, China; College of Animal Science and Technology, Northwest A&F University, Yangling, Shaanxi 712100, China; College of Animal Science and Technology, Northwest A&F University, Yangling, Shaanxi 712100, China; College of Animal Science and Technology, Northwest A&F University, Yangling, Shaanxi 712100, China; College of Animal Science and Technology, Northwest A&F University, Yangling, Shaanxi 712100, China; College of Animal Science and Technology, Northwest A&F University, Yangling, Shaanxi 712100, China; Shaanxi Institute of Zoology, Xi'an, Shaanxi 710032, China; Department of Thyroid and Breast Surgery, Shenzhen Luohu Hospital Group Luohu People’s Hospital (Third Affiliated Hospital of Shenzhen University), Shenzhen, Guangdong 518000, China; Luonan Science and Technology Bureau, Shangluo, Shaanxi 726000, China; College of Forestry, Northwest A&F University, Yangling, Shaanxi 712100, China; College of Animal Science and Technology, Northwest A&F University, Yangling, Shaanxi 712100, China

**Keywords:** seasonal breeding, gut microbiota, testis, polyamine metabolism

## Abstract

Seasonal breeding is an important adaptive strategy for animals. Recent studies have highlighted the potential role of the gut microbiota in reproductive health. However, the relationship between the gut microbiota and reproduction in seasonal breeders remains unclear. In this study, we selected a unique single food source animal, the flying squirrel (*Trogopterus xanthipes*), as a model organism for studying seasonal breeding. By integrating transcriptomic, metabolomic, and microbiome data, we comprehensively investigated the regulation of the gut-metabolism-testis axis in seasonal breeding. Here, we demonstrated a significant spermatogenic phenotype and highly active spermatogenic transcriptional characteristics in the testes of flying squirrels during the breeding season, which were associated with increased polyamine metabolism, primarily involving spermine and γ-amino butyric acid. Moreover, an enrichment of *Ruminococcus* was observed in the large intestine during the breeding season and may contribute to enhanced methionine biosynthesis in the gut. Similar changes in *Ruminococcus* abundance were also observed in several other seasonal breeders. These findings innovatively revealed that reshaping the gut microbiota regulates spermatogenesis in seasonal breeders through polyamine metabolism, highlighting the great potential of the gut-testis axis in livestock animal breeding and human health management.

## Graphical Abstract

**Graphical Abstract:**
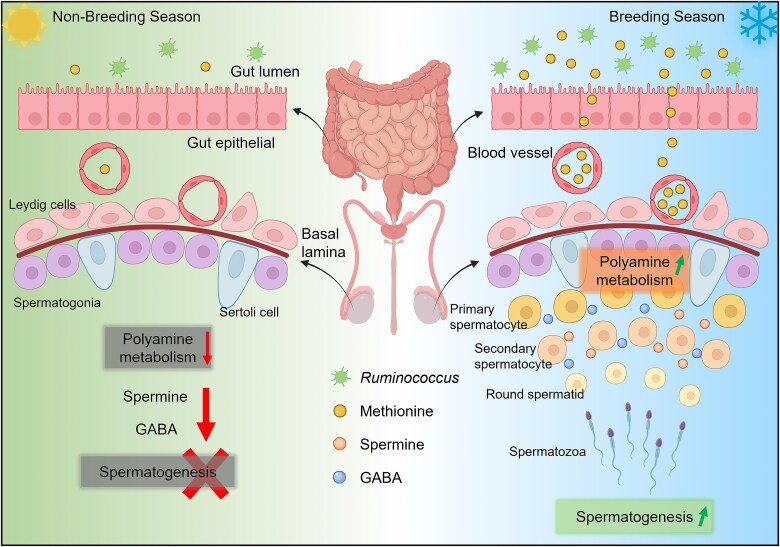

## Introduction

Seasonal breeding is a phenomenon in which organisms alter their reproductive behaviors in response to environmental fluctuations [1]. This strategy provides clear advantages to animals, as they regulate the delivery and upbringing of their offspring during moderate climate seasons (such as spring and summer) to enhance the survival chances of their progeny. Conversely, during less favorable periods (such as winter), they adopt behaviors aimed at survival rather than reproduction [2]. For seasonal breeders, there are dramatic changes in gonadal size during the breeding season (BS), particularly in birds where it can increase more than 100-fold [3]. Despite decades of research, the mechanisms underlying seasonal reproduction in vertebrates remain only partially understood. A critical factor widely recognized in previous studies is that photoperiodic changes regulate melatonin secretion by the pineal gland, thereby influencing the hypothalamic–pituitary-gonad (HPG) axis [4], which is also the subject of extensive research. A recent study demonstrated that removal of the pineal gland does not fully abolish seasonal responses in Siberian hamsters [5], indicating the involvement of other factors in the regulation of seasonal reproduction.

The gut microbiome is a complex ecosystem within the human gastrointestinal tract. It contains thousands of species of bacteria, viruses, fungi, and protozoa [6]. It not only plays a crucial role in digestion and metabolism but also participates in various behavioral and physiological processes of the host, including immune function [7], reproduction [8], social interactions [9, 10], as well as pathological development [11–13]. Previous studies have suggested that the gut microbiota dysbiosis is strongly associated with male infertility [14, 15]. Various internal and external factors, such as diet [8, 16], illness [17, 18], and hormones [19, 20], can disrupt the balance of gut microbiota and subsequently impact reproductive ability. For example, in a metabolic syndrome model, a decreased abundance of *Ruminococcaceae_NK4A214_group* was shown to induce abnormal vitamin A metabolism, which further led to disrupted spermatogenesis [8]. Conversely, a healthy gut microbiota could promote secretion of anti-inflammatory factors (such as IL-10, SCFAs, and dihydrotestosterone) to protect the testicular immune microenvironment and maintain testicular function [20]. Other studies have indicated that changes in photoperiod can also influence the composition of gut microbiota. In Siberian hamsters, individuals exposed to long-day photoperiods exhibited significant changes in gut microbiota composition compared to those exposed to short-day photoperiods, including increased *Lachnospira* abundance and decreased *Sharpea* abundance [21, 22]. The pineal gland and melatonin also play an important role in regulating gut microbiota [22, 23]. Previous studies have found that melatonin supplementation can increase *Lactobacillus* abundance [24], while the *Enterobacter aerogenes* possesses a melatonin-sensitive circadian clock that responds to pineal and gastrointestinal melatonin [25]. Given these findings, the relationship between gut microbiota and reproductive mechanism in seasonal breeders becomes an intriguing but still under-researched subject.

Flying squirrels (*Trogopterus xanthipes*) are small, arboreal, folivorous mammal endemic to China. They have unique dietary types and niches, living in treetops of coniferous forests and primarily consuming leaves of Chinese arborvitae (*Platycladus orientalis*), supplemented with occasional pine nuts and acorns [26]. Moreover, flying squirrels are typical seasonal breeders, producing only one litter per year during a BS that spans from late December to January. Compared to other wild animals, flying squirrels maintain a more consistent diet throughout the year, while compared to domesticated animals, they are more responsive to variations in BSs. Therefore, the combination of their single diet and seasonal breeding characteristics makes flying squirrels an ideal natural model for investigating the relationship between seasonal breeding and gut microbiota.

Our study aimed to investigate the association of the gut microbiota with seasonal breeding and explore the underlying gut-testis axis mechanism. We chose the flying squirrels in BS and nonBS (nBS) as our model. Here, we expect that (i) flying squirrels exhibit more pronounced reproductive characteristics in BS; (ii) transcriptome and metabolome reveal potential genetic and metabolic changes in testis; (iii) the composition of gut microbiota between BS and nBS flying squirrels differs significantly and is associated with reproduction performance; and (iv) the *Ruminococcus* in the LI influences the reproductive phenotype in BS by regulating spermine and γ-amino butyric acid (GABA) metabolism in testis. Overall, our results suggest that the bacteria-metabolite-testis axis was closely associated with seasonal breeding, and targeting *Ruminococcus* might be a promising strategy in regulating animal seasonal reproduction.

## Materials and methods

### Animal models and sample collection

All animal procedures were approved by the Animal Ethics Committee of Northwest A&F University (AAEWV-NWSUAF-DK2023023), and conducted in accordance with the Care and Use of Laboratory Animals at Northwest A&F University. A total of 12 adult male wild flying squirrels were trapped in the Qinling Mountains near Shangluo (Supplementary Fig. S1). Of these, six were captured between June 2023 and August 2023 (nBS) and six between November 2023 and January 2024 (BS) (area: 33° 56′ N to 34° 0′ N, 110° 8′ E to 110° 13′ E). All flying squirrels were adult males (2–3 years of age) with similar body weight. The captured animals were temporarily kept in a family owned flying squirrel farm located in Beikuanpingzhen, Shangluo city, Shaanxi Province of China (33° 54′ 21.3″ N, 110° 8′ 59.5″ E). (In certain villages of the Chinese province of Shaanxi, flying squirrels are farmed on ethnobiological or herbological grounds.) The flying squirrels were anesthetized and subsequently decapitated to obtain bilateral testes, bilateral epididymides, and contents from both the large and small intestines (SIs). Testes and epididymides weights were recorded. The epididymides were used for the determination of sperm. Samples were fixed in 4% paraformaldehyde or placed at −80°C for further analysis.

The breeding and nonbreeding seasonal fecal samples of flying squirrels, chipmunk (*Tamias sibiricus*), Chinese forest musk deer (*Moschus berezovskii*), and domestic dogs (*Canis lupus familiaris*) were previously collected by our laboratory. Fresh fecal samples were collected from the rectum of each animal and placed into a box with dry ice. Next sent to the laboratory and stored at −80°C.

### Sperm analysis

The cauda epididymides were isolated and sliced into pieces in 1 ml of M2 medium (Sigma, M7167, USA). Then incubated at 37°C in an atmosphere containing 5% CO_2_ for 10 min to release sperm into suspension. The suspended sperm was collected and imaged using a computer-assisted sperm analyzer (CASA; HVIEW, Shenzhen, China).

### H&E staining

H&E staining was performed using a standard protocol as previously described [27]. Briefly, testicular and epididymal tissues were fixed in 4% paraformaldehyde and routinely embedded in paraffin after being placed on a shaker at room temperature overnight. The tissues were then sliced into 5 μm thick sections and stained with H&E. Finally observed by a light microscope (Nikon Ni-U, Tokyo, Japan). A minimum of six randomly selected fields of view per section underwent morphological analysis, which was conducted independently by three investigators blinded to the experimental conditions.

### Immunofluorescence and immunohistochemical staining

The staining procedure was adapted from previous studies [28, 29]. Briefly, the fixed testes were dehydrated and sliced into 5 μm sections. Next, rehydration and protein blocking were performed, followed by incubation with the primary antibodies. Afterward, the sections were incubated with the appropriate secondary antibodies. Both positive areas and fluorescence density were measured using ImageJ software. The primary detection antibodies were anti-Kit (Abcam; ab317843), anti-SYCP3 (Novus Biologicals, NB300-232), anti-SOX9 (HUABIO; ET1611-56), and anti-AKAP3 (Invitrogen; PA5-99257).

### RNA isolation and qPCR

RNA isolation and qPCR were conducted following the standard procedures previously reported [27, 30]. Briefly, the total RNA of each sample was extracted using Trizol reagent (Invitrogen, USA). The RNA was then reverse-transcribed into cDNA using a Color Reverse Transcription Kit (EZBioscience, Suzhou, China), followed by qPCR in a Quant Studio 6 Real-Time PCR System (Thermo Fisher Scientific, USA) using the One-Step RT-PCR Kit with SYBR Green (EZBioscience, China). The primer sequences are listed in Supplementary Table S1. Gene expression levels were quantified by normalization of each amplicon to GAPDH.

### Western blotting

Protein samples were extracted from testes using radioimmunoprecipitation assay (RIPA) buffer. The quantification of all proteins was examined using an Enhanced BCA Protein Assay Kit (Beyotime, China). Western blot analysis was then performed following standard procedures. Briefly, using the SDS–polyacrylamide gel electrophoresis (SDS-PAGE) to separate the target proteins from equal total protein, and transferred into PVDF membranes (Millipore Corporation, USA). The membranes were blocked, then incubated with primary and secondary antibodies, followed by visualization using a chemiluminescent reagent. Finally, the density of bands was quantified using the ImageJ software and normalized to β-actin. The antibodies used in this study included SOX9 (HUABIO; ET1611-56), β-actin (4970, CST), and HRP-conjugated anti-rabbit IgG (ab205718, Abcam).

### RNA sequencing

RNA sequencing was performed using testicular tissues from different seasons. RNA extraction, quality verification, library preparation, and sequencing were performed by NovoGene (Beijing, China). Briefly, clean data were obtained by filtering low-quality reads from raw data, and further mapped to the reference genome. Differential expression genes (DEGs) were analyzed using DESeq2, the parameters based on fold change (FC) > 2 and *P* < 0.05 as the threshold to screen DEGs. Gene Ontology (GO) and Kyoto Encyclopedia of Genes and Genomes (KEGG) enrichment analysis of the DEGs were performed. STRING analysis was used to predict the interaction of genes and proteins.

### Untargeted metabolomics study

The sample preparation procedures were performed according to our previously reported protocols [27]. The testicular tissue samples were ground in liquid nitrogen, and methanol–water (7:3, *v*/*v*) was added to eliminate proteins (1 ml for 30 mg sample). Then, ~800 μl supernatant was collected after 15 min ultrasonic treatment and 15 min 12 000 × g centrifuging at 4°C, and dried in a vacuum concentrator at 37°C. Next, the samples were reconstituted in 400 μl methanol–water (1:1, *v*/*v*) by 10 min sonication on ice, followed by a second centrifugation (15 min, 12 000 × g, at 4°C). The supernatant was then transferred to a fresh glass vial for UPLC-Orbitrap-MS/MS analysis.

Metabolomic analyses were conducted using a Dionex UltiMate 3000 UPLC system (Thermo Fisher Scientific, USA), with instrument control, data acquisition, and data analysis carried out through Xcalibur software (version 3.0), as described in previous study [31]. Chromatographic separation was achieved using a C_18_ Hypersil Gold column (100 mm × 2.1 mm, 1.9 μm, Thermo Fisher Scientific), with ultrapure water containing 0.1% formic acid (eluent A) and acetonitrile (eluent B) as the mobile phases, at a flow rate of 0.2 ml/min. The gradient elution program was as follows: 0–3 min, 5%–7% B; 3–5 min, 7%–13% B; 5–15 min, 13%–50% B; 15–18 min, 50%–5% B, followed by a 2-min equilibration at 5% B. The total run time was 20 min, with the column maintained at 35°C and an injection volume of 2 μl. Compound Discoverer 2.1 software (Thermo Fisher Scientific, USA) was used to process raw data to produce data matrix including retention time (RT), mass spectrometry (*m*/*z*), and peak intensity (exclusion criteria: metabolic features with a relative standard deviation >30%). Then the mzCloud and mzVault libraries were searched to identify metabolites.

Principal component analysis (PCA) and partial least squares discriminant analysis (PLS-DA) were performed using the MetaboAnalyst 6.0. The FCs of metabolites were determined by comparing the average peak area levels, and statistical significance was assessed using an unpaired Student’s *t*-test to calculate the *P* value. Differential metabolites were deemed as FC > 2 and *P* < 0.05, and KEGG database was applied to functionally annotate them. Gene Set Enrichment Analysis (GSEA) was performed to enrich metabolites to specific metabolite classes, as described in previous study [32].

### 16S rRNA gene amplicon sequencing

Total DNA of contents from the small and LI was isolated using a QIAamp Fast DNA Stool Mini Kit (51604, QIAGEN) following the manufacturer’s instructions. After determining the quality of DNA by NanoDrop 2000 (Thermo Fisher Scientific, USA) and 1% agarose gel, V3–V4 region of 16S rRNA genes was amplified using the universal primers (F: 5′-CCTAYGGGRBGCASCAG-3′; R: 5′-GGACTACNNGGGTATCTAAT-3′). PCR products were purified using a GeneJET Gel Extraction Kit (Thermo Fisher Scientific, USA) and sequencing libraries were constructed using a NEB Next Ultra II FS DNA PCR-free Library Prep Kit (NEB, USA) following the manufacturer’s instructions and added the index codes. Then NovaSeq platform (Illumina, USA) was used to sequence the library, and 250 bp paired-end reads were generated by NovoGene (Beijing, China). FLASH (v. 1.2.71) was used to merge the pair-end reads, then data were quality filtered using QIIME (v. 1.9.1) to obtain high-quality clean tags and remove all chimeric sequences. Next the high-quality sequences were de-noised using the DADA2 plugin (V.3.11) in QIIME2. Finally, each amplicon sequence variant (ASV) was annotated based on the SILVA database (version 138).

Rarefaction curves, alpha-diversity (Shannon, and Simpson), beta-diversity (principal coordinates analysis [PCoA] and nonmetric multidimensional scaling [NMDS]) based on Bray–Curtis, and evolution tree were performed on NovoMagic (https://magic.novogene.com/). Linear discriminant analysis (LDA) effect size (LEfSe) was processed with the default setting of LDA score ≥ 4 using LEfSe software. Microbial functions were predicted using PICRUSt2 and Tax4fun against KEGG databases.

### Bacterial quantification

The fresh fecal samples were isolated and stored in sterile tubes. Then fecal DNA were extracted in an isolated, low-contamination and controlled environment, using the QIAamp Fast DNA Stool Mini Kit (51604, QIAGEN). Next using the QuantiFast SYBR Green PCR kit (EZBioscience, China) to quantitative PCR the DNA to measure the bacterial 16S rRNA levels. The primer for *Ruminococcus* (F: 5′-GTGTCGTGAGATGTTGGGTTAAGT-3′; R: 5′-AGTGCTCTTGCGTAGCAACTAAAG-3′) and total bacterium (F: 5′-ACTCCTACGGGAGGCAGCAG-3′; R: 5′-ATTACCGCGGCTGCTGG-3′) were used. Results were expressed as relative expression with universal 16S rRNA serving as the internal control.

### Statistical analysis

Unless otherwise noted, data analysis and visualization were performed using GraphPad Prism 6.0 and SPSS Statistics 26 software, and the results are presented as means ± standard error of the mean (SEM). Spearman’s correlations were computed using the R software (v3.6.3) and visualized using OmicStudio tools (available at https://www.omicstudio.cn). A significance level of *P* < .05 indicated statistical significance, and *P* < .1 represented a trending difference. Statistical analysis methods and biological replicates for each experiment are indicated in the corresponding figure legends.

## Results

### Different testicular characteristics between the breeding season and nonbreeding season groups in different seasons

Considering the seasonal changes affect the food source available to animals, which leads to significant alterations in their gut microbiota. Therefore, we selected a unique animal model of a single food source (flying squirrels) for our follow-up study. We trapped a total of 12 adult wild flying squirrels in the Qinling Mountains near Shangluo, 6 during the winter (BS) and 6 during the summer (nBS), all within a single year. There were no significant differences in body weight between the squirrels captured in each season (Supplementary Fig. S1).

**Figure 1:**
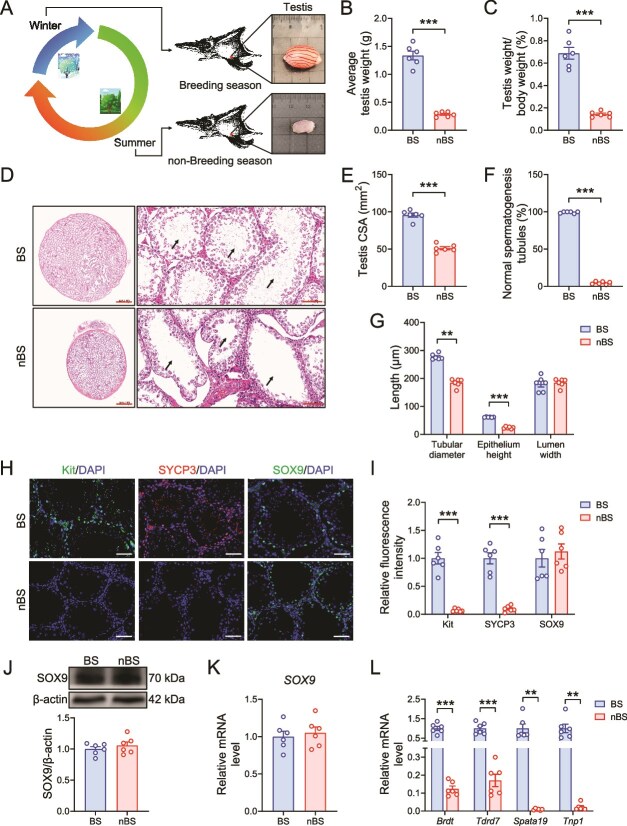
The testicular characteristics of flying squirrels in different seasons. (A) The representative testicular images of flying squirrels in BS and nBS. (B) Average testis weight. (C) Ratio of testis weight/body weight. (D) Representative images of H&E staining in testis sections, scale bar = 2 mm (left) and 100 μm (right). (E) The testis CSA. (F) The proportion of normal spermatogenesis tubules in testis. (G) The tubular diameter, epithelium height, and lumen width of the testis in H&E staining. The representative images (H) and quantification (I) of Kit, SYCP3, and SOX9 immunofluorescent (IF) staining in sections of testicular tissue, scale bar = 100 μm. The SOX9 protein (J) and *SOX9* mRNA (K) expression in testis. (L) The mRNA expressions of *Brdt, Tdrd7, Spata19*, and *Tnp1* in testis. *n* = 6 for each group. All data are presented as means ± SEM. Statistical significance was determined by unpaired Student’s *t*-test. ^**^P < .01 and ^***^P < .001.

To identify the reproductive phenotypes, we detected the testicular and epididymis characteristics between BS and nBS of flying squirrels. The results showed that seasonal changes significantly affected the testicular characteristics of flying squirrels. The squirrels had larger testis during BS (Fig. 1A–C), but there were no changes in epididymis (Supplementary Fig. S2A–C). Further, H&E staining revealed an increase in the cross-sectional area (CSA), tubular diameter, and epithelium height of testis during BS (Fig. 1D, E, and G). Spermatogenesis exhibited significant changes in both testis and epididymis. During the BS, the spermatogenesis tubules were filled in the testis (Fig. 1F), and numerous mature sperm were observed in the cauda epididymis (Supplementary Fig. S2D and E), which were not observed in the nBS testis. Immunofluorescent staining also determined the expressions of some proteins for the reproductive-related genes. And both the c-Kit (marked spermatogonial differentiation) and SYCP3 (marked spermatocytes) were nearly not expressed in the testis during nBS, except for SOX9 (marked Sertoli cells) (Fig. 1H and I). The protein and mRNA expression of SOX9 also showed no significant difference between BS and nBS (Fig. 1J and K). Furthermore, the expression of mRNAs involved in late spermatogenic events (including *Brdt*, *Tdrd7*, *Spata19*, and *Tnp1*) exhibited similar trends during the transition between BS and nBS (Fig. 1L). These results indicate significant seasonal variations in the testicular characteristics of flying squirrels.

### Different transcriptomic characteristics in testis in different seasons

**Figure 2:**
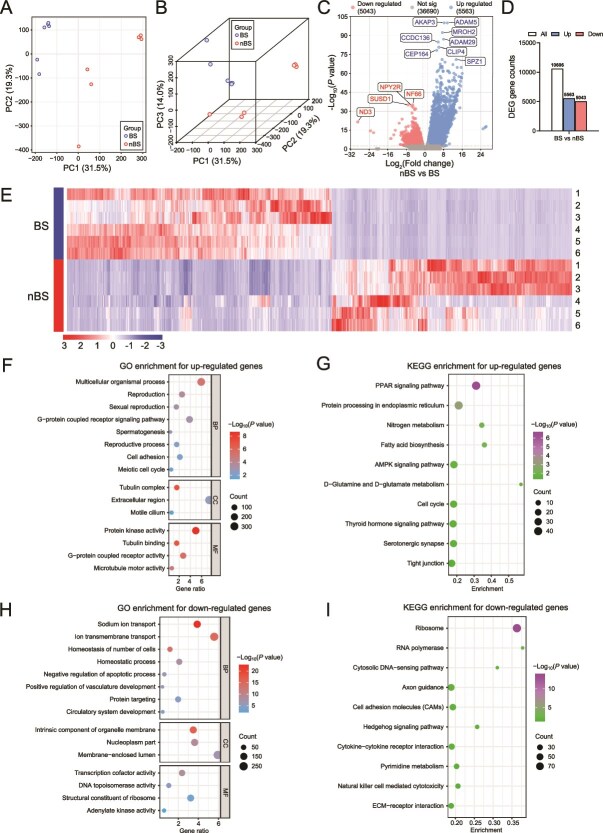
The RNA-Seq analysis of testis during different seasons. The PCA (A) and 3D PCA (B) score plot for all samples. (C) The volcano plot of DEGs. (D) DEG genes counts. (E) The Heatmap of the DEGs. The GO (F) and KEGG (G) enrichment analysis for up-regulated DEGs. The GO (H) and KEGG (I) enrichment analysis for down-regulated DEGs. *n* = 6 for each group.

To further investigate the underlying mechanisms of spermatogenesis in the testis during different seasons. Testicular tissues from BS and nBS flying squirrels were collected for RNA-Seq analysis. The PCA and 3D PCA score plot showed a distinct separation existed in the genes between two groups (Fig. 2A and B). Compared to the nBS group, 5563 genes were up-regulated and 5043 genes were down-regulated in the BS group (Fig. 2C and D). Cluster analysis also revealed significant differences between the BS and nBS groups (Fig. 2E). Then, the up-regulated genes were analyzed using GO analysis; the genes were enriched in reproduction and spermatogenesis-related pathways (Fig. 2F). KEGG enrichment revealed that PPAR signaling pathway, nitrogen metabolism, AMPK signaling pathway, and D-glutamine and D-glutamate metabolism were the main pathways in the spermatogenesis during BS (Fig. 2G). However, the down-regulated genes tended to enrich in homeostatic-related pathways (Fig. 2H). The hedgehog signaling pathway, pyrimidine metabolism, and ECM–receptor interaction were the major factors in the process (Fig. 2I).

**Figure 3:**
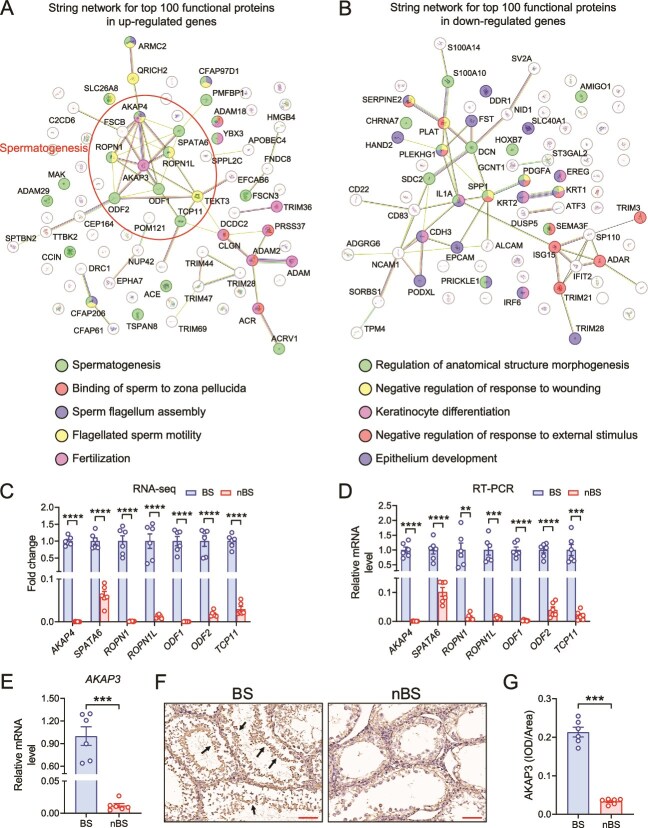
The changes in the reproductive season alter the transcriptomic characteristics in testis. Schematic of the prediction of protein–protein interaction for top 100 up-regulated (A) and down-regulated (B) genes. (C) The transcripts of spermatogenesis-related genes. (D) Relative mRNA expression of spermatogenesis-related genes. The AKAP3 expression in testis were determined by RT-PCR (E) and immunohistochemistry (F and G), scale bar = 100 μm. *n* = 6 for each group. All data are presented as means ± SEM. Statistical significance was determined by unpaired Student’s *t*-test. ^**^P < .01, ^***^P < .001, and ^****^P < .0001.

The protein–protein interaction of the top 100 up-regulated and down-regulated genes was predicted (Fig. 3A and B). The string network indicated that most of the up-regulated DEGs were associated with the spermatogenesis and its related pathways. And down-regulated DEGs were associated with the regulation of anatomical structure morphogenesis, epithelium development, and others. The spermatogenesis-related genes including *AKAP4*, *SPATA6*, *ROPN1*, *ROPN1L*, *ODF1*, *ODF2*, and *TCP11* (Fig. 3C), which were also validated by RT-PCR analysis (Fig. 3D). We next determined the AKAP3 expression in testis, which was strongly interacting with the spermatogenesis-related genes in string network. Both mRNA and protein expression of AKAP3 were significantly increased in BS (Fig. 3E–G). These findings implied that the testis exhibits active spermatogenic transcriptional characteristics during BS.

### Different metabolic characteristics in testis in different seasons

**Figure 4:**
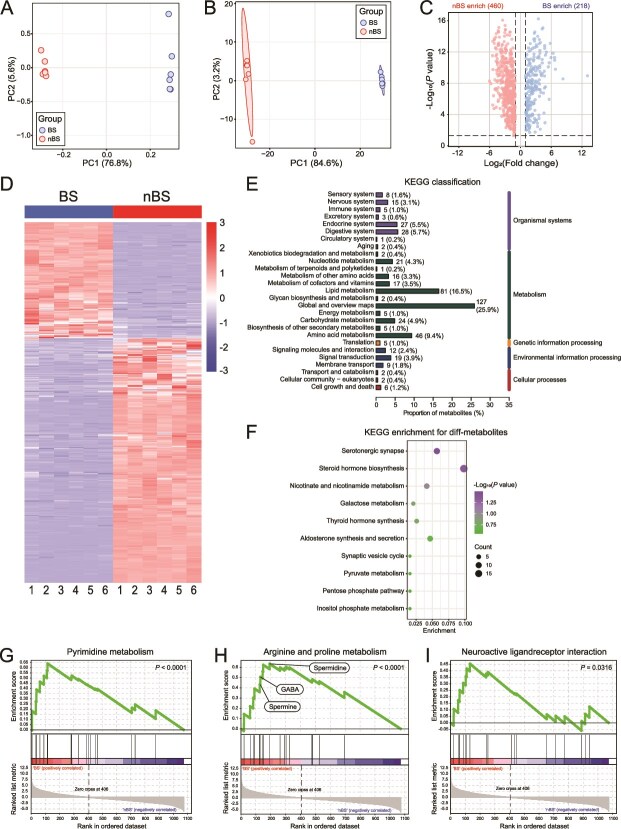
The metabolism changes in testis during different seasons. (A) The PCA score plot. (B) The PLS-DA score plot. (C) The volcano plot of differential expression metabolites. (D) Heatmap of the differential metabolites. The KEGG classification (E) and enrichment analysis (F) of differential metabolites. (G–I) The GSEA analysis for metabolites. *n* = 6 for each group.

After finding the significant transcriptomic differences between testis in BS and nBS groups, we next set out to explore the metabolic differences in the testicular microenvironment. Testicular metabolites were determined by nontargeted metabolomic sequencing to investigate the response mechanisms of metabolites in seasonal breeding. A total of 1074 metabolites were found, and nearly half of them were lipids and lipid-like molecules (Supplementary Fig. S3A). Data were analyzed by PCA (Fig. 4A and B) and PLS-DA (Supplementary Fig. S3B and C) analyses, and both of the score plots showed that the BS and nBS groups could be clearly separated. The data suggested that changes in BSs influenced metabolic profiles in the flying squirrel’s testis. There were 678 significantly altered metabolites in BS vs nBS, 460 of which were highly expressed in nBS group, and 218 in BS group (Fig. 4C and D). The KEGG database was further used to enrich and analyze these metabolites. These differentially expressed metabolites were classified (Fig. 4E), and they mainly involved pathways such as serotonergic synapse, steroid hormone biosynthesis, thyroid hormone synthesis, and aldosterone synthesis and secretion (Fig. 4F and Supplementary Fig. S3D and E). GSEA analysis revealed only three pathways were significantly enriched, including pyrimidine metabolism (*P* < .0001), arginine and proline metabolism (*P* < .0001), and neuroactive ligand receptor interaction (*P* < .05) (Fig. 4G–I). Among them, arginine and proline metabolism were closely associated with reproductive processes. And many metabolites involved in spermine metabolism (spermidine, GABA, and spermine) were core enriched in BS group. We also conducted a targeted analysis of some spermatogenesis-related metabolites (such as androstenedione, testosterone, and corticosterone), all of them displayed expression trends consistent with the GSEA-enriched metabolites (high level in BS group) (Supplementary Fig. S3F–K).

**Figure 5:**
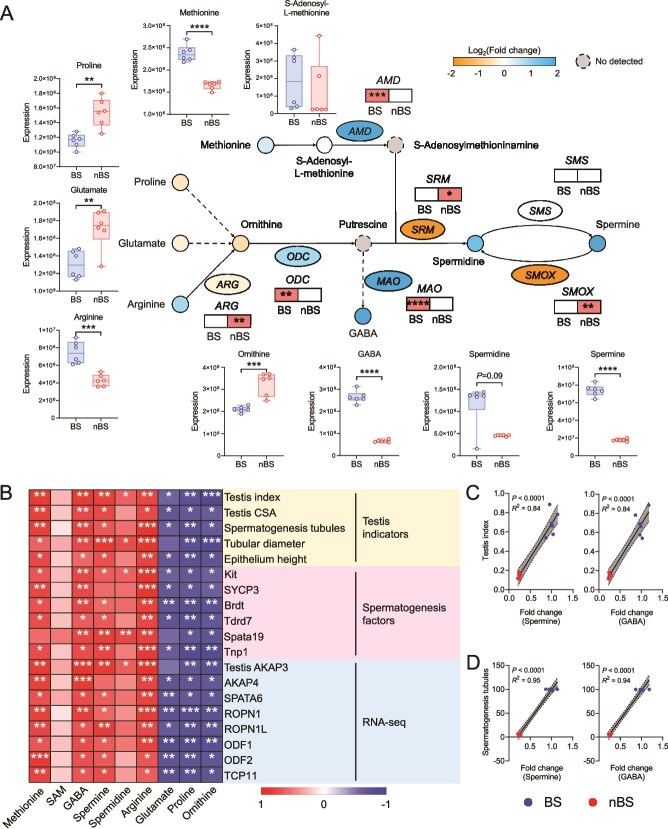
The abnormal polyamine metabolism in testis during nBS. (A) Pathway of polyamine metabolism in testis. (B) Spearman’s correlation analysis of metabolome and transcriptome. (C) and (D) Two-tailed Pearson’s correlation coefficient analysis of testicular characteristics and differential metabolites. *n* = 6 for each group. All data are presented as means ± SEM. Statistical significance was determined by unpaired Student’s *t*-test for A. ^**^P < .01, ^***^P < .001, and ^****^P < .0001.

We further focused on analyzing spermine metabolism in the testis (Fig. 5A and Supplementary Fig. S4). The expression of *ODC* and *AMD*, which participate in polyamine biosynthesis, was significantly increased during BS. And the mRNA levels of *MAO* had similar trends, suggesting the up-regulated synthesis of GABA during BS. Conversely, *SMOX* and *SRM*, both regulators of spermidine synthesis, were decreased. During BS, the levels of substrates for polyamine synthesis (proline, glutamate, and ornithine) were reduced in testis. Spermidine and spermine were increased. Furthermore, correlation analysis revealed that methionine, GABA, spermine, and arginine levels were significantly positively related to testis and spermatogenesis-related factors (Fig. 5B). Linear regression analysis further verified the positive relationship between spermine, GABA levels, and testis index and spermatogenesis tubules (Fig. 5C and D). Taken together, these data indicated that the different BSs caused distinct metabolic characteristics in the testis, and implied that spermine and GABA metabolism may play an important role in testis spermatogenesis during BS.

### Seasonal reshaping of the gut microbiota

**Figure 6:**
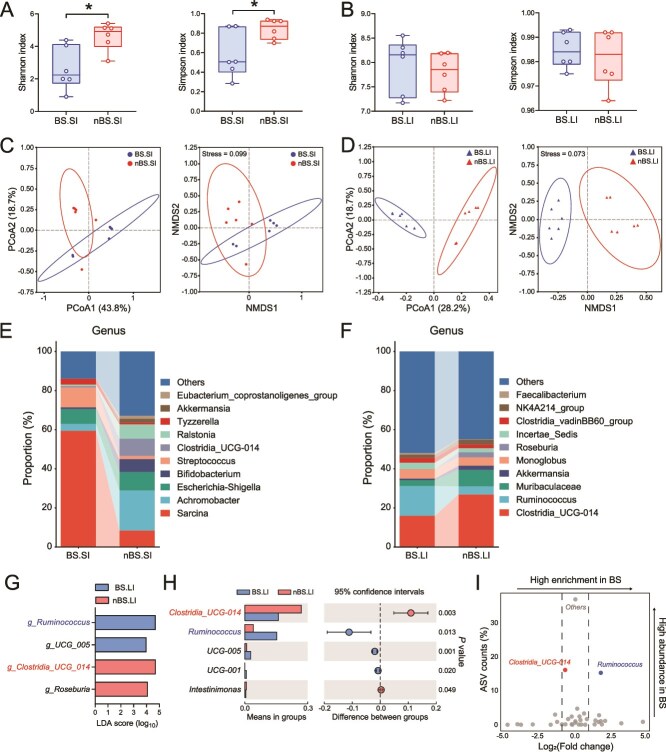
Profiling of the gut microbiota was altered in different seasons. The alpha diversity (Shannon and Simpson indices) of the gut microbiota in SI (A) and LI (B). The PCoA (left) and NMDS plot (right) based on Bray–Curtis between BS and nBS groups in SI (C) and LI (D). Average relative abundances of predominant taxa at genus level in SI (E) and LI (F). (G) The most differentially abundant taxa between BS and nBS groups in LI identified by LDA effect size. Only LDA threshold of ≥4 taxa are shown. (H) The relative abundances of *g_Ruminococcus, g_UCG_005*, *g_Clostridia_UCG_014*, and *g_Roseburia* in LI. (I) Volcano plot for the relative abundance distribution of microbial ASVs in LI. Each symbol represents one bacterial taxon. *n* = 6 for each group. Data were represented as means ± SEM or means with 95% confidence interval (CI). *P* values were analyzed by unpaired Student’s *t* test. ^*^P < .05.

Given the important role of the gut microbiota in regulating hosts metabolism and its interactions with the physiology and disease of hosts [33, 34], and considering the spatial heterogeneity of gut microbiota [35]. We next collected gut microbiota samples from the SI and LI of flying squirrels, respectively. We then sequenced the 16S rRNA gene amplicons isolated from the gut microbiota in different seasons. Rarefaction curve and rank abundance curve analyses indicated that the data were reliable and sequencing depth was sufficient to represent the bacterial diversity (Supplementary Fig. S5A–E). BSs influenced the alpha diversity of microbiota in the SI (decreasing both the Shannon and Simpson indices), but no changes were observed in the LI (Fig. 6A and B). We also found distinct clustering of microbiota composition in the LI between BS and nBS groups using PCoA and NMDS plot based on Bray–Curtis, whereas it is not significantly in SI (Fig. 6C and D). Taxonomic variations at phylum, family, and genus levels indicated unique microbial compositions between BS and nBS in both the SI and LI (Supplementary Fig. S5F and G and Fig. 6E and F). The evolution tree of TOP100 microbiota in genus level further indicated that the SI bacteria such as *Sarcina*, *Streptococcus*, and LI bacteria such as *Ruminococcus*, were enriched in BS (Supplementary Fig. S6A and B). Moreover, LEfSe analysis identified three core differential bacterial taxa in SI and four in LI at genus level (Fig. 6G and Supplementary Fig. S6C–E). Although the abundance comparison of predominant genera showed that the SI bacterium *g_Sarcina* was significantly enriched in BS, it was not identified by LEfSe analysis (Supplementary Fig. S6F). For LI bacteria, *g_Ruminococcus* and *g_Clostridia_UCG-014* were significantly enriched in BS and nBS, respectively (Fig. 6H and Supplementary Fig.S6G). The further volcano plot flagged *g_Ruminococcus* as the most enriched and representative bacteria in the LI (Fig. 6I). These findings indicate that changes in BSs led to the remodeling of gut microbiota, with the LI bacterium *g_Ruminococcus* potentially playing a crucial role in this process.

### Large intestine microbiota reshape was critically associated with the reproductive changes in breeding season

**Figure 7:**
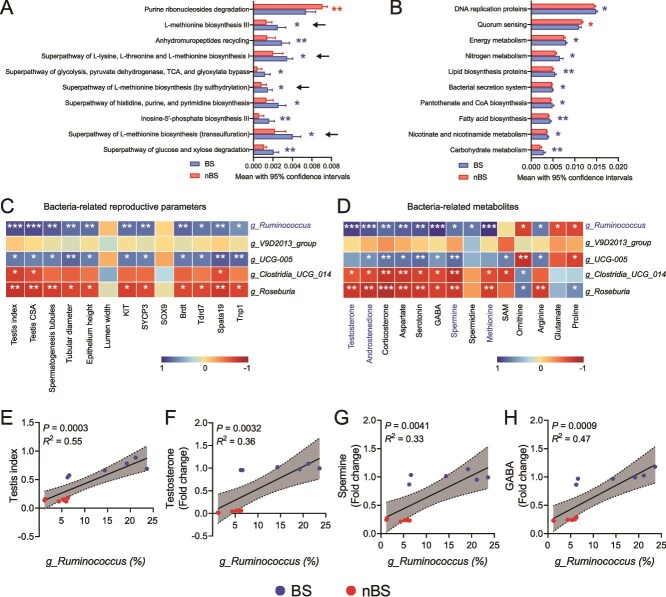
Microbial function prediction of large intestinal microbiota and their relationships with reproductive phenotypes and metabolites. Relative abundance of pathway (top 10) in LI predicted by PICRUSt2 (A) and Tax4fun analysis (B). Spearman’s correlation coefficient analysis between the differential bacteria and reproductive parameters (C), and differential metabolites (D). (E–H) Two-tailed Pearson’s correlation coefficient analysis of abundance of *Ruminococcus* and testis index, relative level of testosterone, spermine, and GABA. *n* = 6 for each group. Data were represented as means with 95% CI and statistical significance was determined by unpaired Student’s *t*-test for A and B. ^*^P < .05 and ^**^P < .01.

In recent years, there has been increasing evidence supporting a relationship between gut microbiota and testicular function [8, 16]. Building on the above results, we further focused on the microbiota in LI. Microbial functions were predicted using PICRUSt2 and Tax4fun analysis. The PICRUSt2 analysis revealed that methionine biosynthesis-related pathways were enriched in BS (marked by black arrow) (Fig. 7A), and methionine was also involved in polyamine synthesis. Tax4fun analysis also revealed that various nutrients biosynthesis pathways, including lipids, fatty acids, and carbohydrate, were enriched in BS (Fig. 7B). Subsequently, we tested the correlation between the key microbiota in LI and reproductive parameters (Fig. 7C), and differential metabolites (Fig. 7D). Correlation analysis suggested that *g_Ruminococcus* may play a beneficial role in stimulating spermatogenesis, regulating the reproductive-related metabolites synthesis, and up-regulating spermine and GABA metabolism during BS. Linear regression analysis also found that the abundance of *g_Ruminococcus* was positively correlated with the testis index, testosterone, spermine, and GABA levels (Fig. 7E–H). Taken together, our results suggest that gut microbiota is closely associated with the regulation of seasonal breeding, with *g_Ruminococcus* potentially influencing spermine and GABA metabolism in testis, thereby affecting the spermatogenic phenotype during BS.

### Abundance of intestinal *g_Ruminococcus* in different seasonal breeding animals

**Figure 8:**
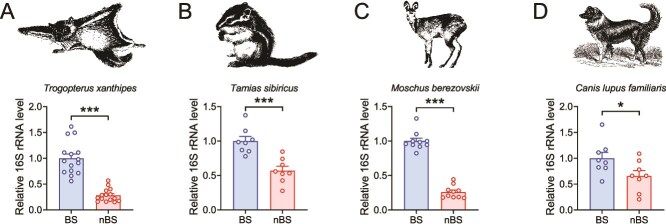
The relative abundance of *g_Ruminococcus* in gut microbiota of different seasonal breeding animals in different BSs. (A) Flying squirrels (*Trogopterus xanthipes*), *n* = 15. (B) Chipmunks (*Tamias sibiricus*), *n* = 8. (C) Chinese forest musk deer (*Moschus berezovskii*), *n* = 10. (D) Dog (*Canis lupus familiaris*), *n* = 8. All data are presented as means ± SEM. Statistical significance was determined by unpaired Student’s *t*-test. ^*^P < .05 and ^***^P < .001.

To further assess whether the abundance of *g_Ruminococcus* exhibits seasonal variation in other seasonal breeding animals, we collected fecal samples from a range of species with similar reproductive patterns. This approach aimed to determine if the observed changes in *g_Ruminococcus* abundance during the BS represent a common phenomenon across different species. We evaluated four seasonal breeding animals: flying squirrels (*Rodentia*), chipmunks (*T. sibiricus*, *Rodentia*), Chinese forest musk deer (*M. berezovskii*, *Ruminantia*), and dog (*C. lupus familiaris*, *Mammalia*). As anticipated, a higher abundance of *g_Ruminococcus* was observed in the gut of animals during the BS (Fig. 8A–D). These were consistent with our above results.

## Discussion

The photoperiod is widely recognized as the key regulator of seasonal breeding in animals [36], but it seems not the only factor regulating seasonal breeding [5]. Considering the close association between gut microbiota and various physiological functions, our findings support a hypothesis that gut microbiota may also play a role in regulating the reproductive function of seasonal breeders. In this study, we selected a unique animal model of a seasonal breeder with a single food source, thereby minimizing the impact of food source variability on gut microbiota during seasonal changes. Firstly, we evaluated the differences in reproductive characteristics of flying squirrels between BS and nBS. The results showed a notable elevation of spermatogenesis in testis during BS, whereas it was almost complete disappearance in nBS. Mechanistically, the testis in BS showed up-regulated spermine and GABA metabolism, which was strongly correlated with the abundance of *g_Ruminococcus* in LI. These findings suggested that the reshaping of gut microbiota is closely associated with regulating spermatogenesis in seasonal breeding; spermine and GABA metabolism contributed a pivotal role in the gut-testis axis involved in seasonal breeding regulation. This study also hints at the potential of *g_Ruminococcus* in managing livestock animal breeding and human health.

Seasonal breeding animals often show significant changes in reproductive phenotype during seasonal transitions. In a previous study, Japanese quail achieved spermatogenesis within 2 weeks after being exposed to long-day conditions [37]. In this study, we observed increased testis weight, active spermatogenesis, and numerous mature sperm in the epididymis during BS, which is consistent with findings from several similar studies [38, 39]. Moreover, various markers were used to further evaluate and verify testicular spermatogenesis. Kit and SYCP3 are markers for the normal process of spermatogonial differentiation and meiosis, respectively [40, 41]. SOX9, which is expressed in Sertoli cells, contributes to maintaining the stability of spermatogenic environment [41]. The genes *Brdt*, *Tdrd7*, *Spata19*, and *Tnp1* are involved in late spermatogenic events [12]. Transcriptome was used to further explore the deeper mechanisms. Data suggested that seasonal changes induce substantial alterations in the transcriptional state of the testis. Functional enrichment analysis showed that reproduction-related pathways were significantly enriched in BS. However, the nBS trends to enrich the homeostasis-related pathways. This finding aligns with observations in other seasonal breeding animals [3, 42]. Both the PPAR signaling pathway and AMPK signaling pathway are known for their roles in antioxidant, anti-inflammatory, and anti-apoptotic in testis [43, 44]. And glutamate, the principal amino acid in testicular fluid [45], plays a critical role in maintaining testicular metabolic homeostasis [46, 47]. And they all enriched in BS. Moreover, during BS, the testes exhibited higher expression and protein levels of the prominent genes affecting spermatogenesis [48–51]. Thus, the spermatogenesis effect in BS may mainly due to the recovery of gene expression. Our results provide evidence that the testes exhibit significant spermatogenic changes during the BS. In contrast, such changes are nearly absent during nBS, with the testis trending to maintain a supportive environment in preparation for subsequent BSs.

During spermatogenesis process, metabolic regulation plays a critical role [12, 41]. Previous studies had demonstrated the essential role of arginine and proline play in male reproduction [52, 53]. Polyamines, synthesized de novo from arginine, porline, and methionine, are also involved in multiple stages of spermatogenesis and sperm motility [54]. ODC and AMD are two key enzymes in endogenous polyamine biosynthesis; they regulate the conversion of ornithine and methionine into polyamines, respectively [54]. And they also correlated with spermatid development and played crucial role in cell survival during early murine development [12]. In our results, the biosynthesis of polyamine, especially spermine, was up-regulated in BS testis. In polyamine metabolism, spermine is synthesized through successive aminopropyl transfer reactions mediated by spermidine synthase (SRM) and spermine synthase (SMS), and can be converted into spermidine by SMOX [54]. Spermine can also be transported from other tissues, such as blood circulation and cecum, via polyamine transport proteins [55]. These might explain that although the mRNA levels of spermine synthesis genes were not increased in BS, the testes still exhibited high spermine level. Another finding was the elevated levels of GABA, which may suggest the activation of the polyamine degradation pathway. GABA is the primary inhibitory neurotransmitter in the mammalian central nervous system. However, it has also been identified in the testis, where it is involved in Leydig cell proliferation, testosterone production, and spermatogenesis [56, 57], as reflected in our results. These all implied that polyamine metabolism, centered on spermine and GABA, is involved in testicular spermatogenesis during BS, highlighting the important roles of spermine and GABA in seasonal breeding.

The effects of gut microbiota on physiology have garnered increasing interest. It is regarded as a metabolic organ that is involved in the regulation of various physiological and pathological processes of the host, such as the nervous system [58], reproductive system [16], and metabolic diseases [8, 13]. Previous studies have demonstrated that defects in spermatogenesis induced by a “Westernized” diet could be improved by restoring the gut microbiome [16, 59], such as *Bacteroides* and *Prevotella* [16]. Exposure to toxins and drugs has also been shown to disrupt testicular development through the gut-metabolism-testis axis [41, 60]. In our study, we observed a significant reshaping of gut microbiota across different seasons, and suggested that LI bacteria *Ruminococcus* may be a core genus for spermatogenesis regulation in BS. *Ruminococcus* was first described in 1974 as a strictly anaerobic bacterium residing in the gut of healthy individuals [61], providing nutrients to the host by degrading and fermenting polysaccharides [62]. In a study about Brandt’s voles, the abundance of *Ruminococcus* was shown to have a significant correlation with reproductive hormones [63]. Similar results also were observed in our study, suggesting its positive role in rodent reproduction. Additionally, in sheep, the disruption of *Ruminococcus* was also demonstrated to correlate with testicular oxidative stress, apoptosis, and testosterone secretion [64]. The depletion of L-methionine biosynthesis and a reduced abundance of *Ruminococcus* species have been implicated in various host diseases [65, 66], including diseases that may lead to reproductive issues, such as obesity. Furthermore, methionine deficiency has been established to induce testicular degradation and a decline in sperm quality [67]. In our study, the microbial function of large intestinal bacteria showed an increased trend in L-methionine biosynthesis during the BS. Moreover, in many other physiological and pathological processes, variations in *Ruminococcus* abundance have been closely associated with changes in spermine and GABA metabolism. In rats on a high-protein diet, both *Ruminococcus* abundance and spermine levels were reduced, accompanied by an increased risk of colonic diseases [68]. Increased levels of *Ruminococcus* and GABA have also been shown to alleviate oxidative stress and inflammation [69, 70]. Combined with our results from metabolomics, these findings provide evidence that *Ruminococcus* regulates spermatogenesis in seasonal breeders through spermine and GABA metabolism in the testes. These findings establish a connection between gut microbiota remodeling and spermatogenesis in seasonal breeders.

Seasonal breeding is a remarkable adaptive feature that enables animals to coordinate their physiological functions to stay in tune with the changing seasons throughout the year [1]. However, it also poses limitations in animal production. Traditionally, hormonal treatments have been considered a classical method, but their use incurs significant cost for environment and human health [71, 72]. Other eco-friendly methods, such as photoperiodic treatments and male effect, also exhibit limitations in terms of efficiency and routine application [42, 73]. Our findings suggest that gut microbiota is closely associated with seasonal breeding, which might have significant implications in livestock animal breeding and human health management. However, some limitations were still suffered in this study. First, seasonal breeding is a complex biological process regulated by multiple pathways [3]. We could not able to distinguish the interaction of microbiota and other factors in seasonal breeding regulation due to our animal models. For example, we also observed changes in sex hormones across different BSs, which are well known to contribute to alterations in the composition of the gut microbiota [74, 75]. Although studies directly investigating the impact of seasonal fluctuations in sex hormones on the microbiome remain limited, but we have to consider the possibility that such fluctuations may also involve in modulating gut microbiota to regulate seasonal breeding. In further studies, useful technologies such as pinealectomized animals, and sterile model organisms combined with microbiota transplantation, could be considered to further elucidate the distinct role of microbiota in seasonal breeding. Another limitation is that numerous factors, including diet, environment, and genetics, significantly influence the gut microbiota profile [76]. Thus, although we validated changes in *Ruminococcus* abundance in some other seasonal breeders, it remains an intriguing challenge to determine whether this regulatory mechanism affects in other species animals. Last, taxonomic identification was achieved only at the genus level in this study. Considering that the functions of bacterial species can vary even within the same genus [77, 78], the isolation and verification of specific strains also will be crucial in future studies. We aimed to gradually address these problems step-by-step, and provide further insights into the mechanistic links between seasonal breeding and gut microbiota, along with their potential applications.

## Conclusions

In conclusion, our study provides insights into the dynamic restructuring of the gut microbiota during seasonal reproduction. We identified that testicular polyamine metabolism is activated during BS, contributing to the upregulation of genes associated with spermatogenesis. Which may largely depend on the increased abundance of the LI bacterium *Ruminococcus* and the enhanced methionine biosynthesis in LI. These findings underscore the intricate interplay between gut microbiota and reproductive physiology, highlighting potential avenues to explore the gut-testis axis for future research into the mechanisms governing seasonal reproductive strategies.

## Supplementary Material

Figure_S1_wraf036

Figure_S2_wraf036

Figure_S3_wraf036

Figure_S4_wraf036

Figure_S5_wraf036

Figure_S6_wraf036

Supplementary_Materials_wraf036

## Data Availability

The raw sequencing data (RNA-Seq and 16S rRNA gene sequencing) reported in this paper have been deposited in the Genome Sequence Archive in National Genomics Data Center, China National Center for Bioinformation / Beijing Institute of Genomics, Chinese Academy of Sciences (GSA: CRA019546 and CRA019563) that are publicly accessible at https://ngdc.cncb.ac.cn/gsa. All data generated or analyzed during the study are included in this article and its supplementary information files.
