# Supplementary figures and images for: Microbiota contribute to regulation of the gut-testis axis in seasonal spermatogenesis
Source: ISME J. 2025 Feb 25;19(1):wraf036. doi: 10.1093/ismejo/wraf036 (PMC11964897; doi:10.1093/ismejo/wraf036)

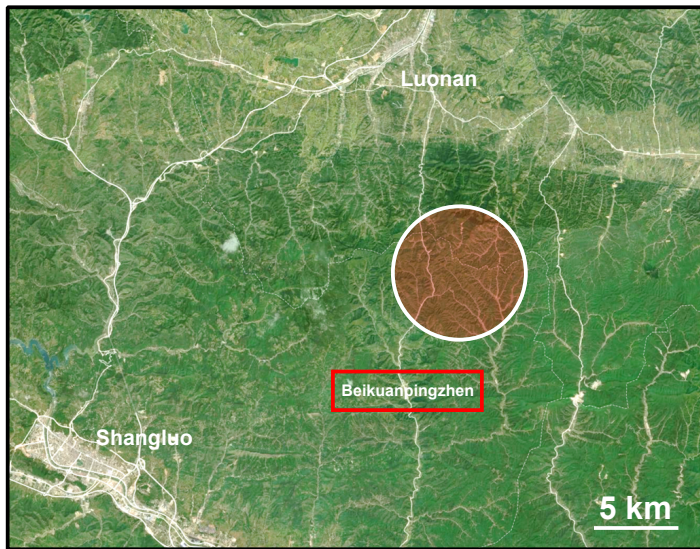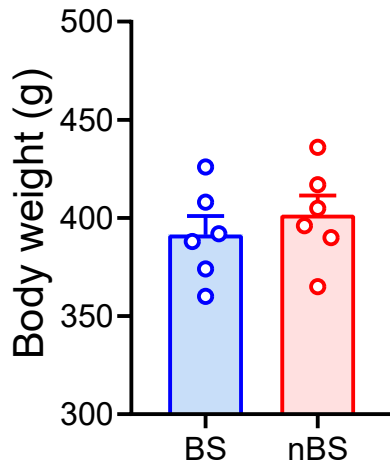

Supplement: Figure_S1_wraf036 [file figure_s1_wraf036.pdf]

**A**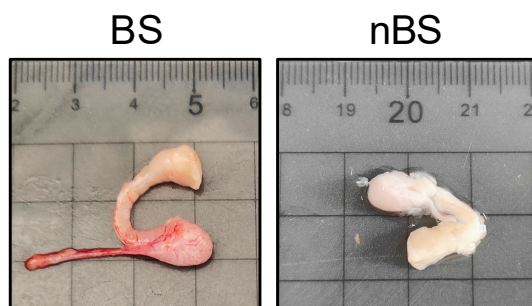**B**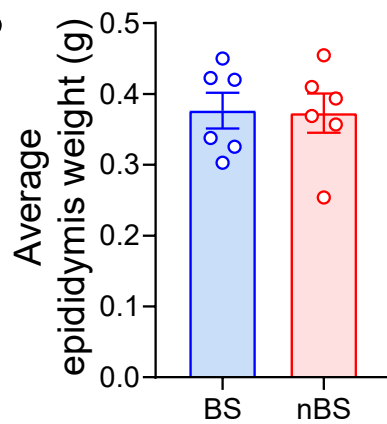**C**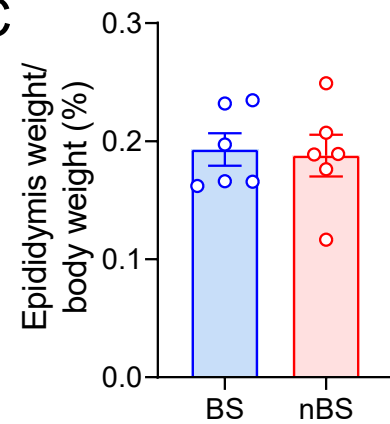**D****BS**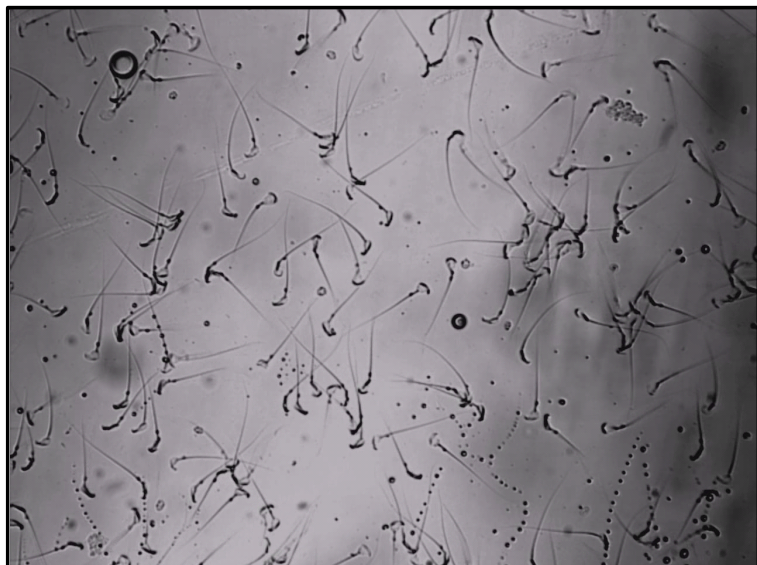**nBS**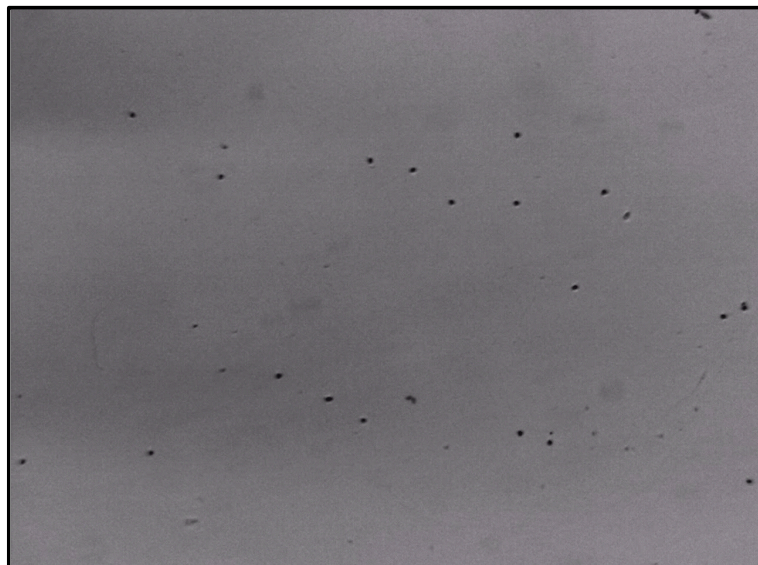**E****BS**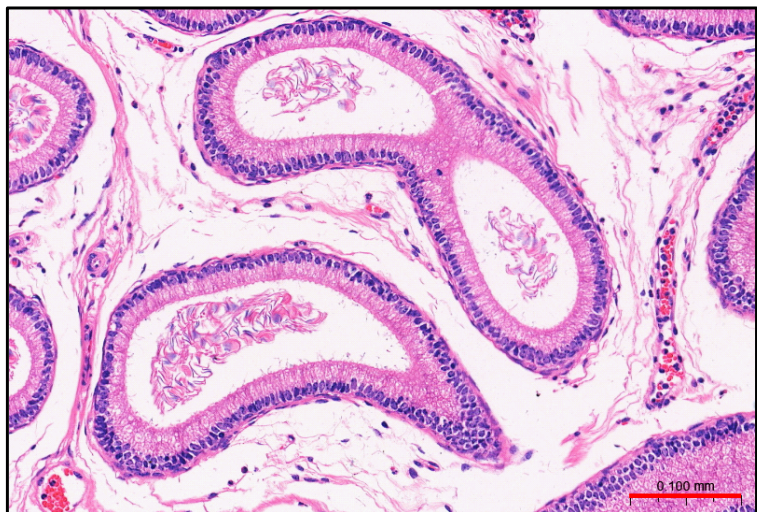**nBS**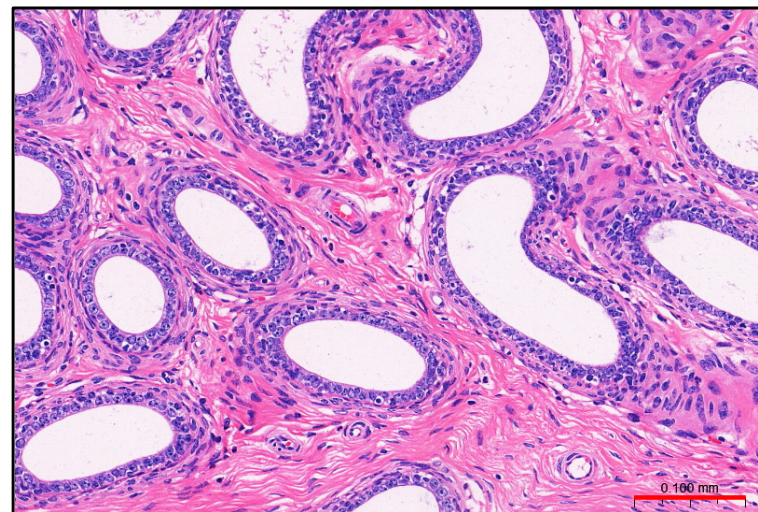

Supplement: Figure_S2_wraf036 [file figure_s2_wraf036.pdf]

**A***AMD*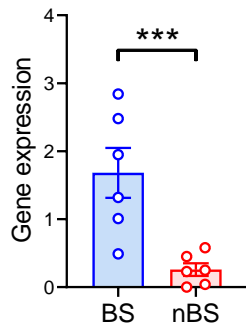**B***ARG*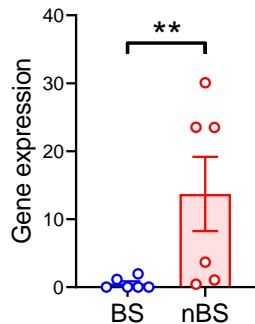**C***ODC*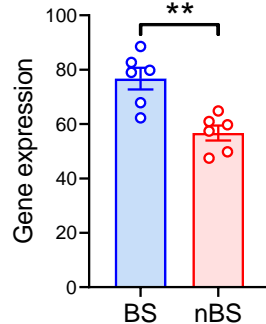**D***MAO*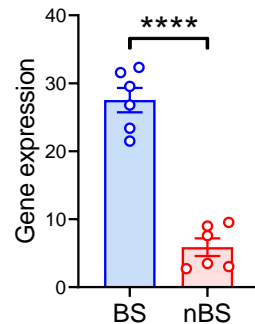**E***SRM*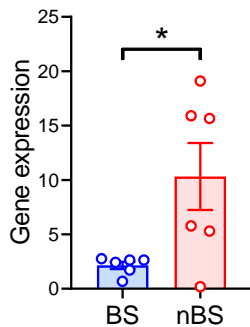**F***SMS*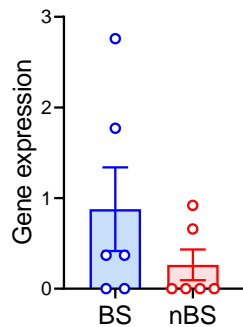**G***SMOX*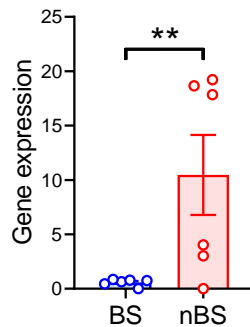

Supplement: Figure_S4_wraf036 [file figure_s4_wraf036.pdf]

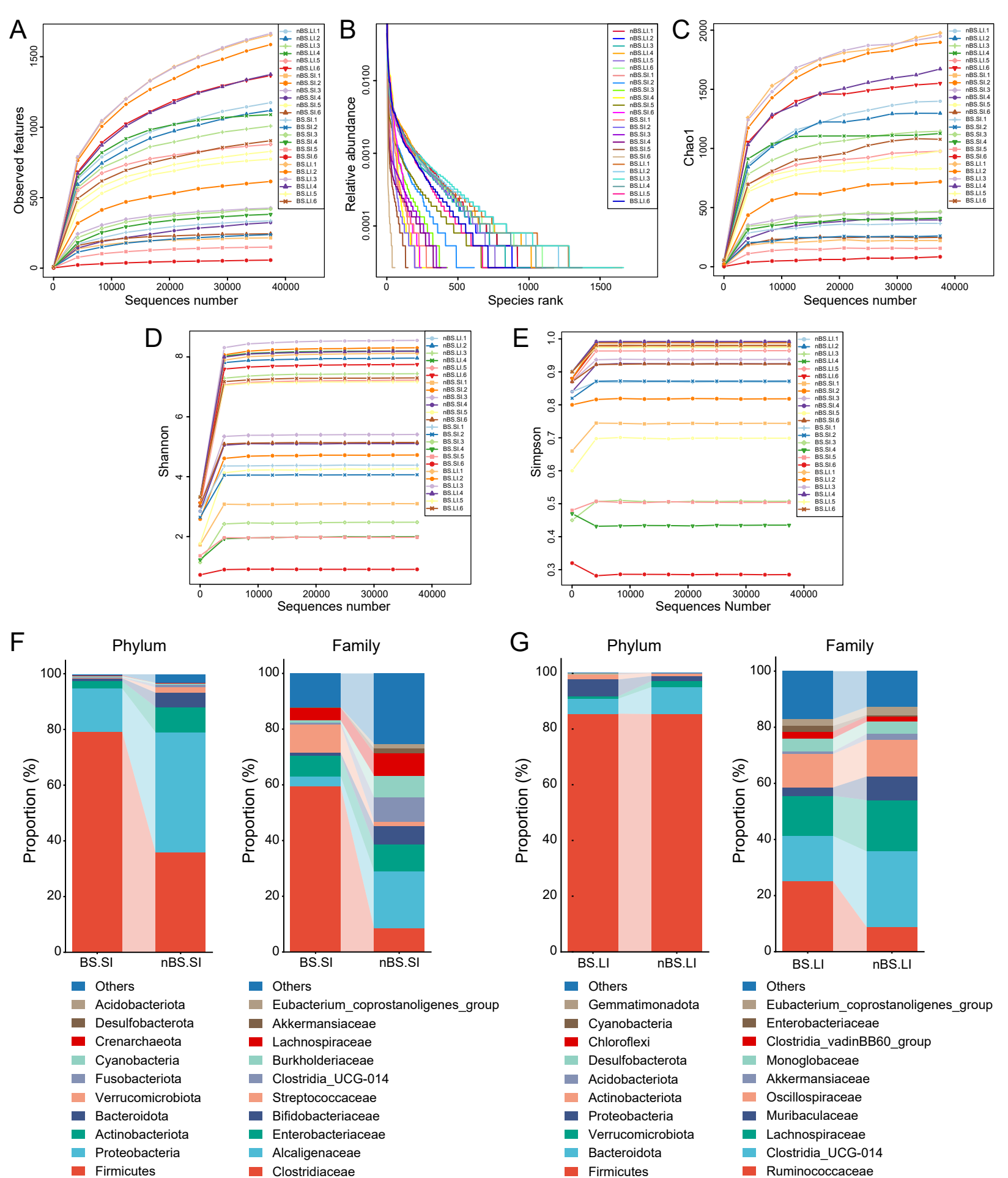

Supplement: Figure_S5_wraf036 [file figure_s5_wraf036.pdf]

A

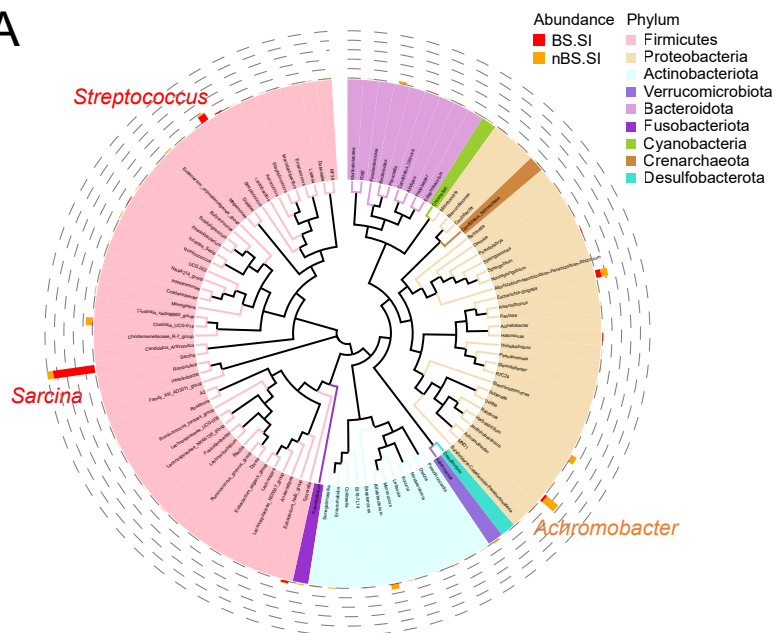

B

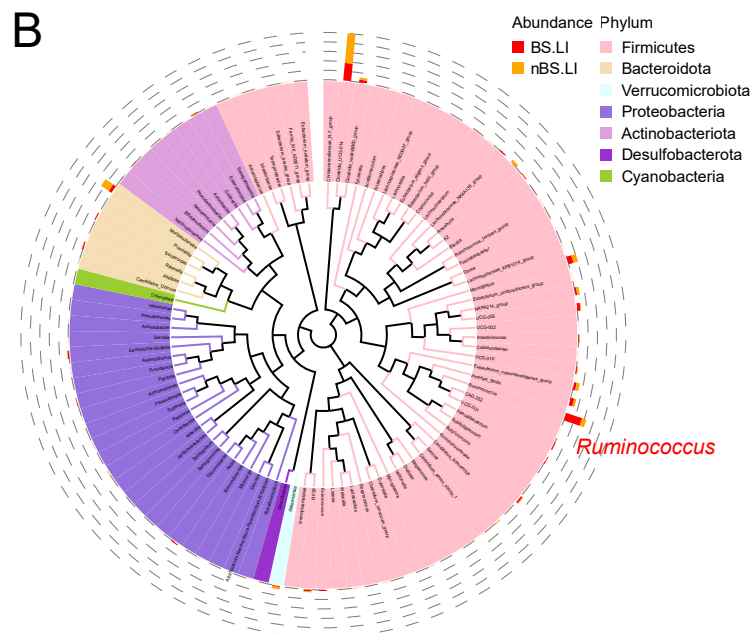

C

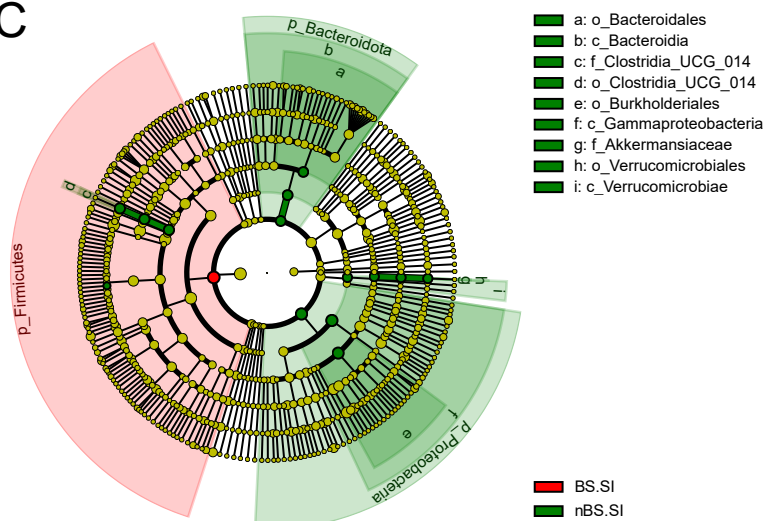

D

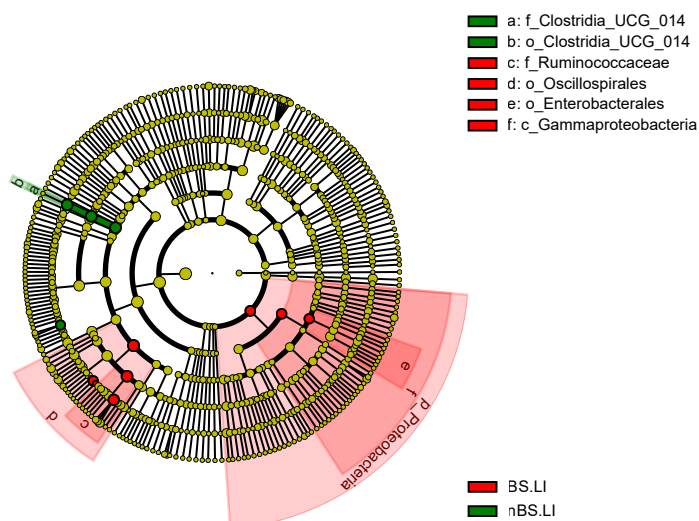

E

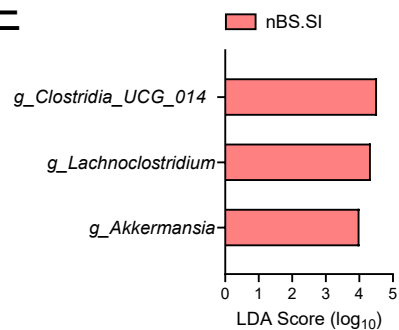

F

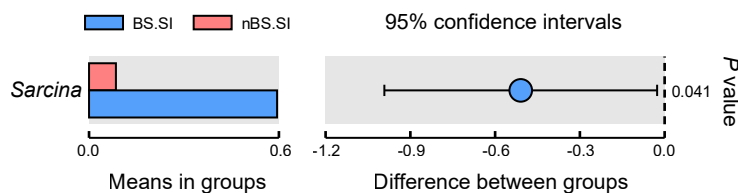

G

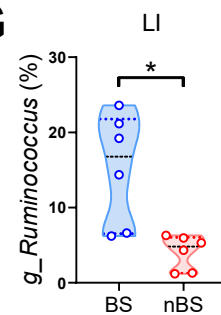

Supplement: Figure_S6_wraf036 [file figure_s6_wraf036.pdf]
