# Supplementary material for: Microbiota contribute to regulation of the gut-testis axis in seasonal spermatogenesis
Source: ISME J. 2025 Feb 25;19(1):wraf036. doi: 10.1093/ismejo/wraf036 (PMC11964897; doi:10.1093/ismejo/wraf036)
Supplement: Figure_S3_wraf036 [file figure_s3_wraf036.pdf]

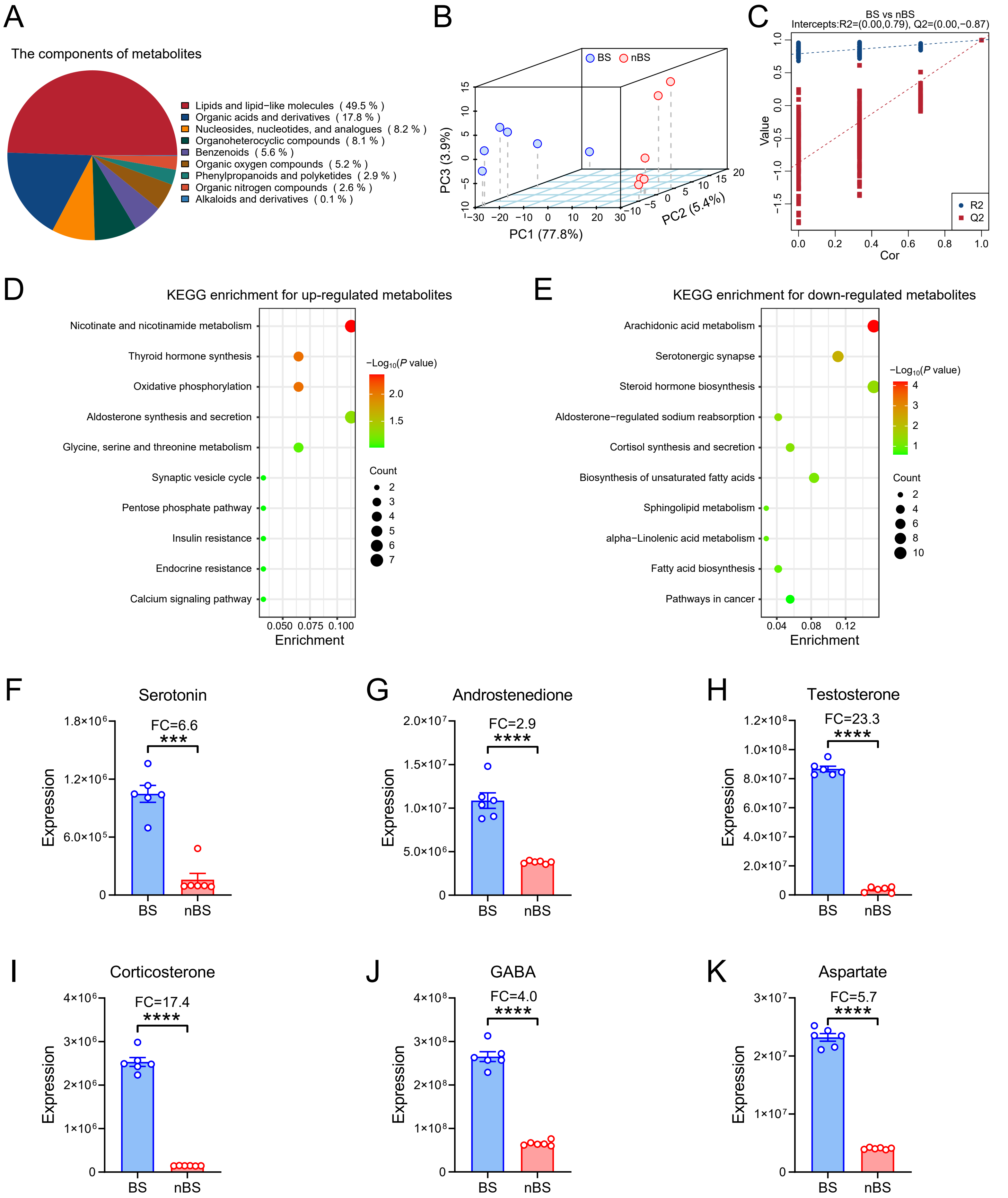

A
The components of metabolites
Lipids and lipid−like molecules ( 49.5 % )
Organic acids and derivatives ( 17.8 % )
Nucleosides, nucleotides, and analogues ( 8.2 % )
Organoheterocyclic compounds ( 8.1 % )
Benzenoids ( 5.6 % )
Organic oxygen compounds ( 5.2 % )
Phenylpropanoids and polyketides ( 2.9 % )
Organic nitrogen compounds ( 2.6 % )
Alkaloids and derivatives ( 0.1 % )
B
BS nBS
PC1 (77.8%)
PC2 (5.4%)
PC3 (3.9%)
C
BS vs nBS
Intercepts:R2=(0.00,0.79), Q2=(0.00,−0.87)
Value
Cor
R2
Q2
D
KEGG enrichment for up-regulated metabolites
Nicotinate and nicotinamide metabolism
Thyroid hormone synthesis
Oxidative phosphorylation
Aldosterone synthesis and secretion
Glycine, serine and threonine metabolism
Synaptic vesicle cycle
Pentose phosphate pathway
Insulin resistance
Endocrine resistance
Calcium signaling pathway
−Log10(P value)
Count
Enrichment
E
KEGG enrichment for down-regulated metabolites
Arachidonic acid metabolism
Serotonergic synapse
Steroid hormone biosynthesis
Aldosterone−regulated sodium reabsorption
Cortisol synthesis and secretion
Biosynthesis of unsaturated fatty acids
Sphingolipid metabolism
alpha−Linolenic acid metabolism
Fatty acid biosynthesis
Pathways in cancer
−Log10(P value)
Count
Enrichment
F
Serotonin
FC=6.6
***
Expression
BS nBS
G
Androstenedione
FC=2.9
****
Expression
BS nBS
H
Testosterone
FC=23.3
****
Expression
BS nBS
I
Corticosterone
FC=17.4
****
Expression
BS nBS
J
GABA
FC=4.0
****
Expression
BS nBS
K
Aspartate
FC=5.7
****
Expression
BS nBS
