# Supplementary material for: Microbiota contribute to regulation of the gut-testis axis in seasonal spermatogenesis
Source: ISME J. 2025 Feb 25;19(1):wraf036. doi: 10.1093/ismejo/wraf036 (PMC11964897; doi:10.1093/ismejo/wraf036)
Supplement: Supplementary_Materials_wraf036 [file supplementary_materials_wraf036.docx]

**Supplementary Materials**

**Microbiota contribute to regulation of the gut-testis axis in seasonal spermatogenesis**

**Zifang Wu^1,‡^, Long Li^1,‡^, Shaoxian Chen^1^, Ye Gong^1^, Yuyan Liu^1^, Tianqi Jin^1^, Yang Wang^1^, Jie Tang^3^, Qian Dong^4^, Bangzhu Yang^5^, Fangxia Yang^2,*^, Wuzi Dong^1,*^**

^1^College of Animal Science and Technology, Northwest A&F University, Yangling, Shaanxi 712100, China

^2^College of Forestry, Northwest A&F University, Yangling, Shaanxi, 712100, China

^3^Shaanxi Institute of Zoology, Shaanxi, 710032, China

^4^Department of Thyroid and Breast Surgery, Shenzhen Luohu Hospital Group Luohu People's Hospital (Third Affiliated Hospital of Shenzhen University), Shenzhen, Guangdong, 518000 China

^5^Luonan Science and Technology Bureau, Shangluo, Shaanxi, 726000, China

*Corresponding authors: Wuzi Dong, College of Animal Science and Technology, Northwest A&F University, No.22 Xinong Road, Yangling, Shaanxi, 712100, China, Email: dongwuzi@nwsuaf.edu.cn; Fangxia Yang, College of Forestry, Northwest A&F University, No.22 Xinong Road, Yangling, Shaanxi, 712100, China, Email: yangfangxia@nwsuaf.edu.cn.

^‡^These authors contributed equally to this work.


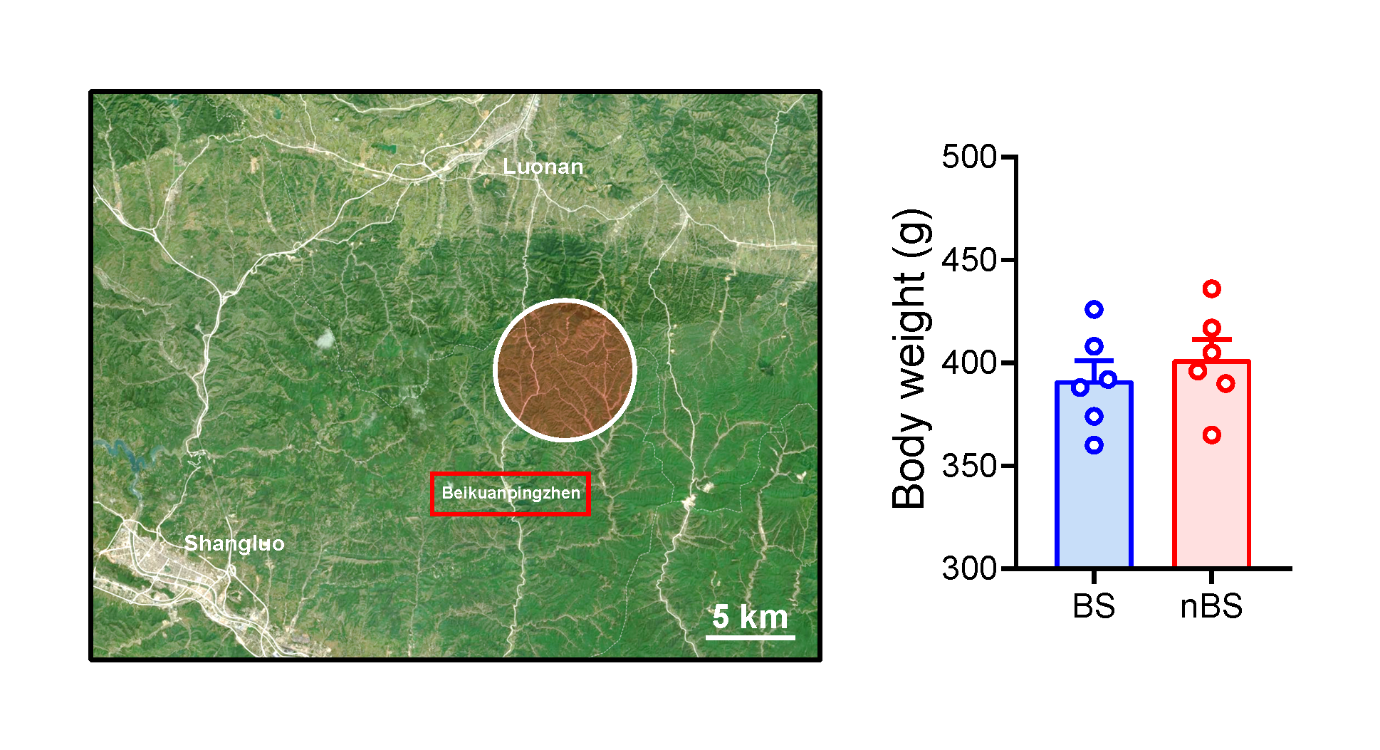


**Figure S1. Geographically trapping locations of *Trogopterus xanthipes* and the body weight.** In winter and summer, flying squirrels were trapped in the Qinling Mountains near Shangluo. The red circle indicates the trapping area, and captured flying squirrels were temporarily housed in captivity at Beikuanpingzhen (marked by the red box). On the right is the body weight of captured flying squirrels.


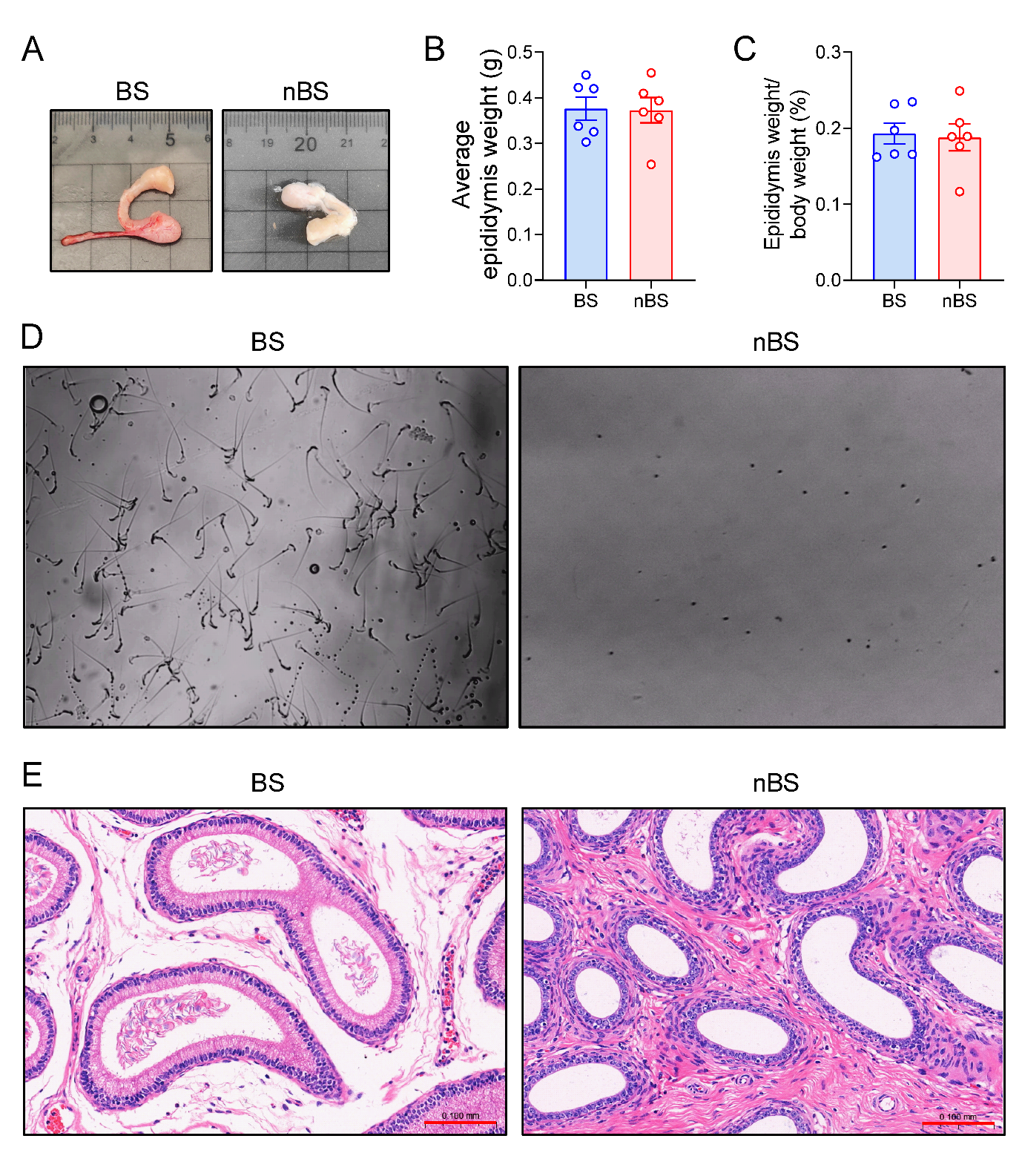


**Figure S2. The epididymis characteristics of** ***Trogopterus xanthipes* in differential season.** (A) The representative images of epididymis. (B) Average epididymis weight. (C) Ratio of epididymis weight/body weight. (D) The representative images of sperm in epididymis. (E) The representative epididymal sections are stained with H&E, scale bar=100 μm. n=6 for each group. All data are presented as means ± SEM. Statistical significance was determined by unpaired Student’s *t*-test.


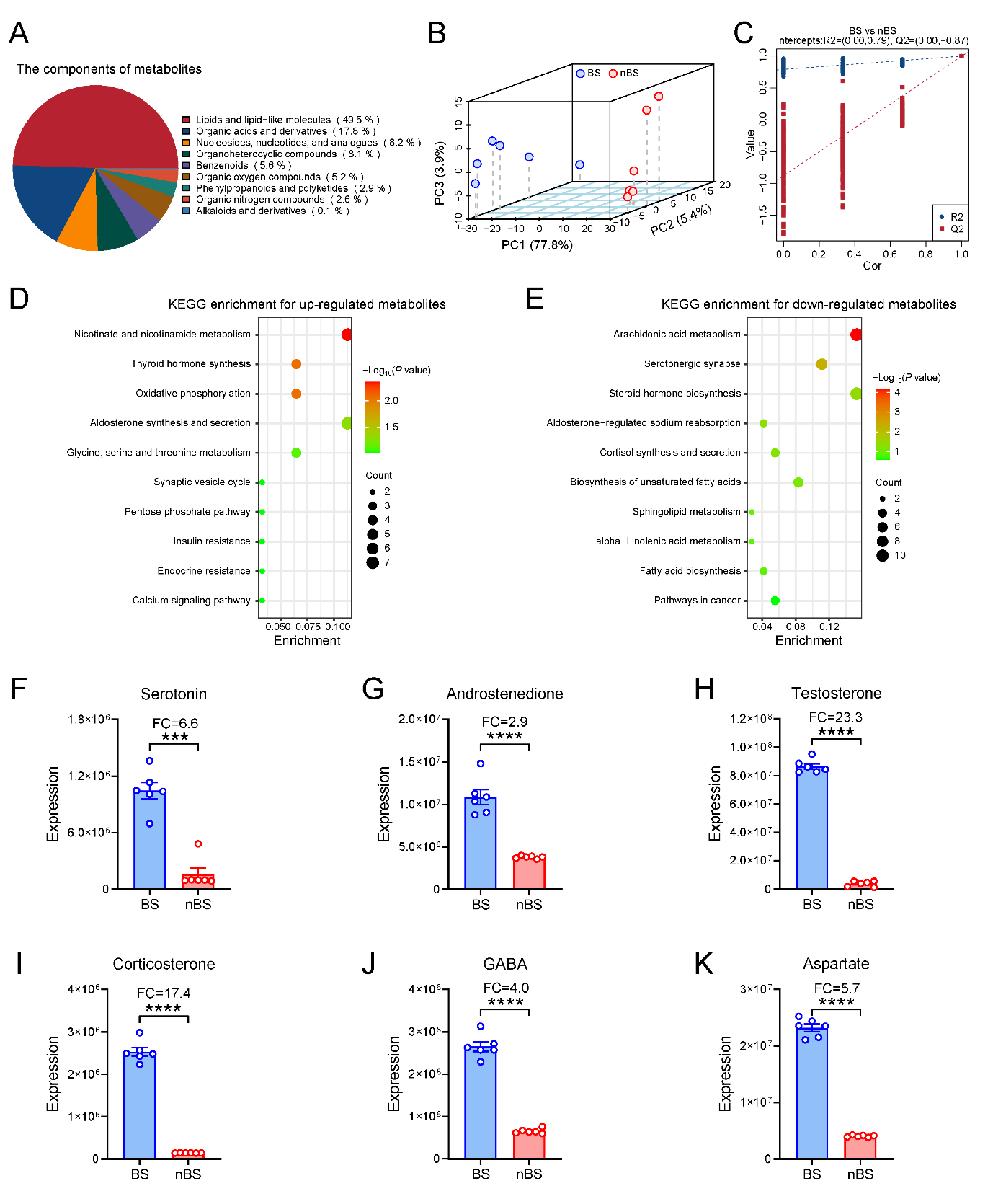


**Figure S3. The metabolism changes in testis during differential season.** (A) The components of metabolites. (B) The 3D PCA score plot. (C) Permutation test. (D-E) The KEGG enrichment analysis of up- and down- regulated metabolites. (F-K) The expression of serotonin, androstenedione, testosterone, corticosterone, γ-aminobutyric acid (GABA), and aspartate in testis. n=6 for each group. All data are presented as means ± SEM. Statistical significance was determined by unpaired Student’s *t*-test. ****P* < 0.001, *****P* < 0.0001.


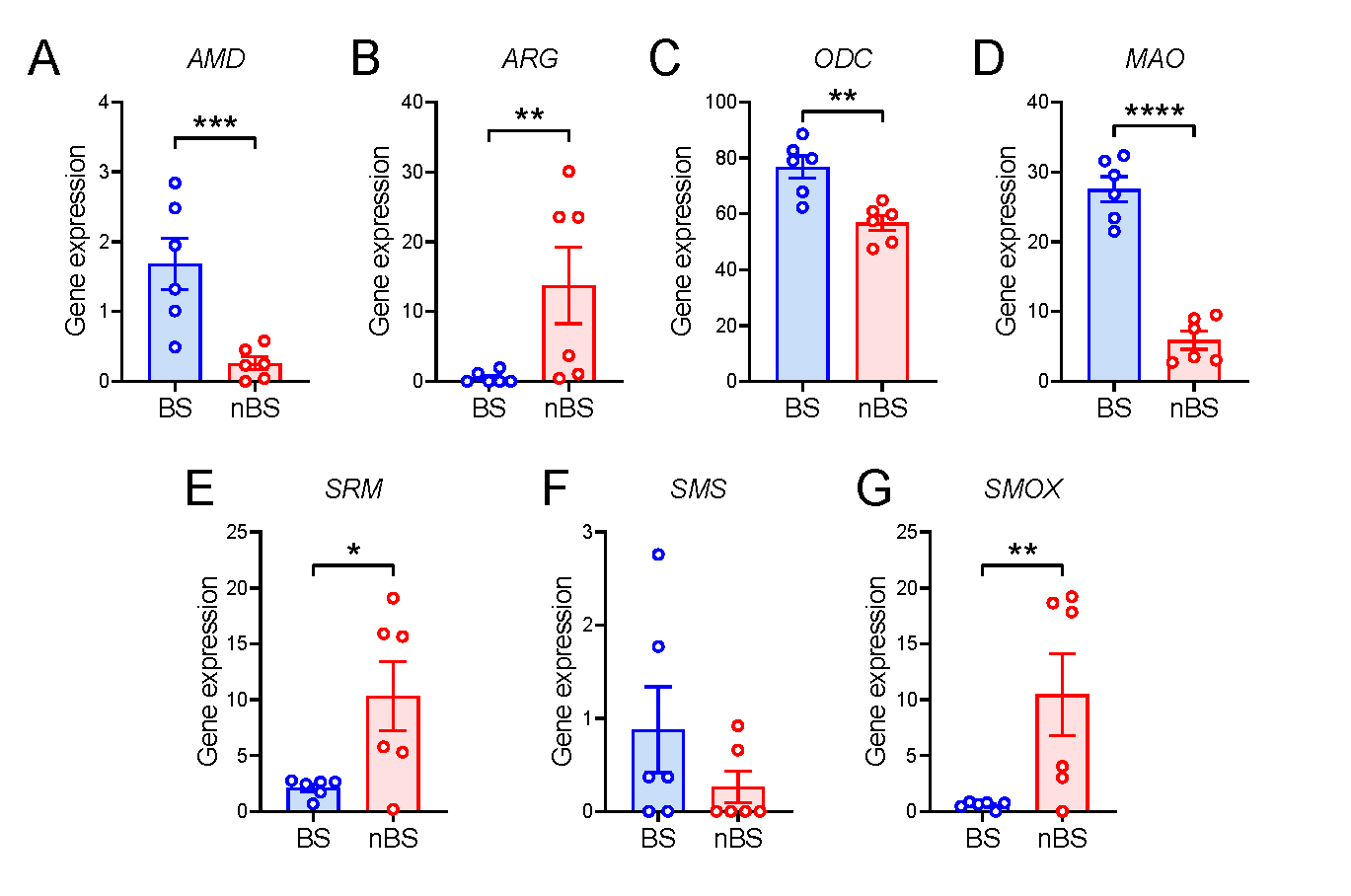


**Figure S4. The transcripts of polyamine metabolism-related genes.** n=6 for each group. All data are presented as means ± SEM. Statistical significance was determined by unpaired Student’s *t*-test. **P*<0.05, ***P* < 0.01, ****P* < 0.001, *****P* < 0.0001.


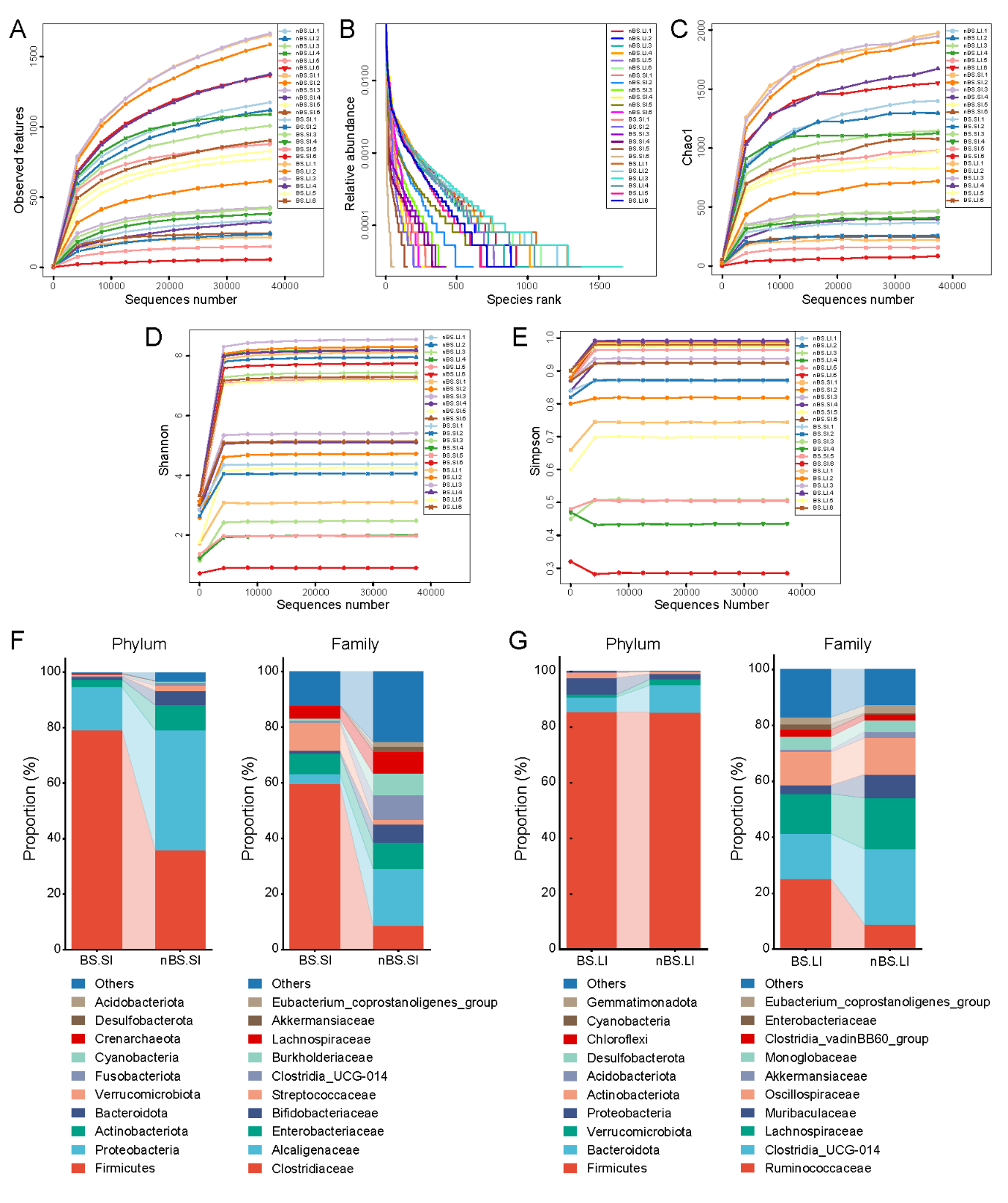


**Figure S5. The rarefaction curve, rank abundance curve, and average relative abundances of predominant taxa of all samples.** (A) observed features. (B) Relative abundance. (C) Chao 1. (D) Shannon. (E) Simpson. (F-G) Average relative abundances of predominant taxa at phylum and family level in small (F) and large (G) intestine.


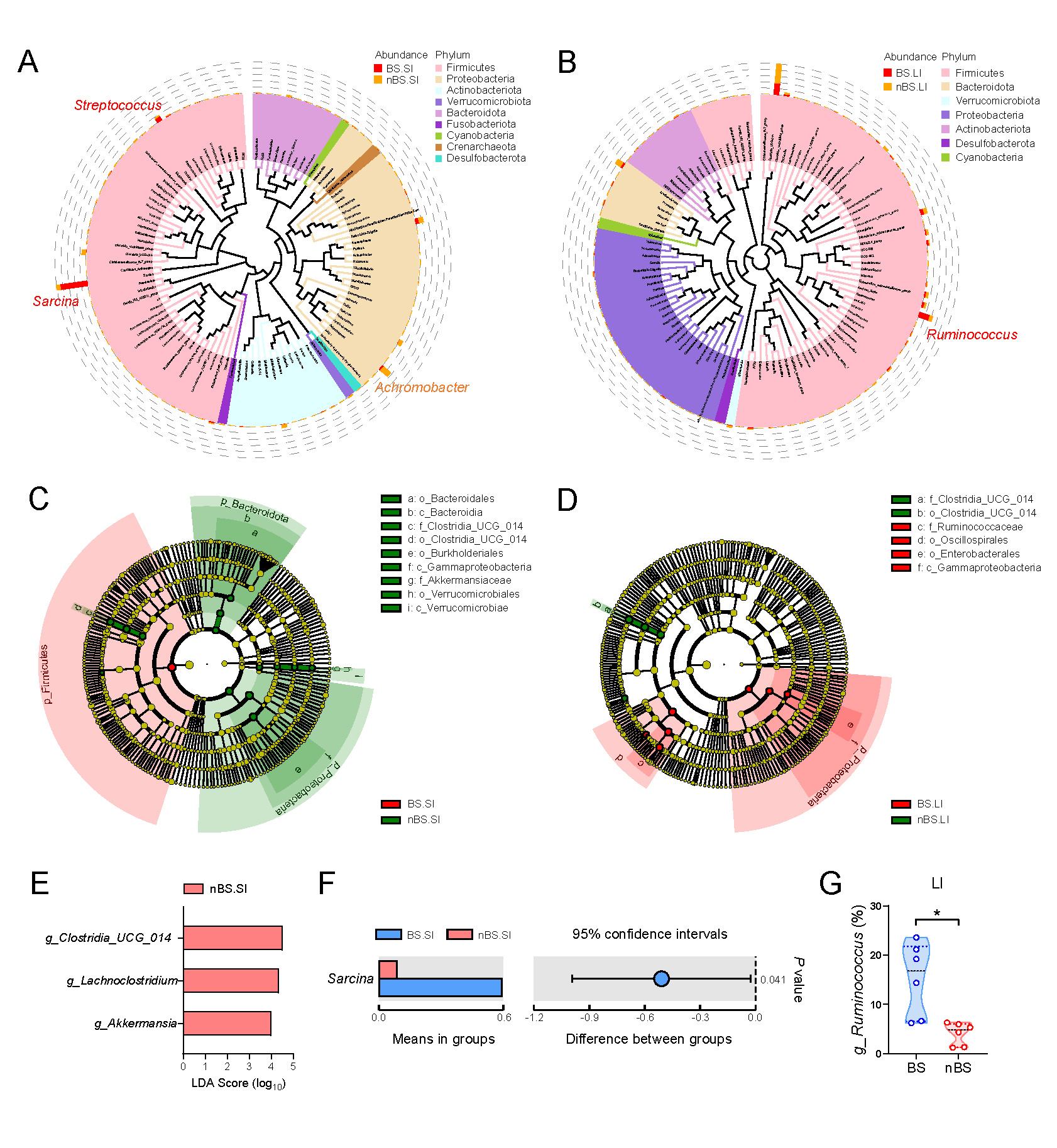


**Figure S6. The changes of gut microbiota in different intestine.** (A-B) The evolution tree of Top100 microbiota (genus level) in small intestine (A) and large intestine (B). (C-D) Cladogram of LEfSe analysis in small intestine (C) and large intestine (D). (E) The LDA score plot of LEfSe analysis in small intestine. (F) The relative abundance of *g_sarcina* in small intestine. (G) The relative abundance of *g_Ruminococcus* in large intestine. n=6 for each group. All data are presented as means ± SEM. Statistical significance was determined by unpaired Student’s *t*-test. **P* < 0.05.

**Table S1.** Primer sequence for qPCR in this study.

| Gene | Sequence (5’-3’) | |
| --- | --- | --- |
| *GAPDH* | Forward: | CAAGGCTGTGGGCAAGGTCATC |
|  | Reverse: | TTCTCCAGGCGGCAGGTCAG |
| *SOX9* | Forward: | AGCACTCGCCGCAGCAG |
|  | Reverse: | AGTTCTGGTGGTCGGTGTAGTC |
| *BRDT* | Forward: | GGGACCTCGGAAATGGATTGACTC |
|  | Reverse: | TTACGGTCTTTGAGAAGCCACAGC |
| *TDRD7* | Forward: | TCCAACAGCAGGTTTAGCCCAAAG |
|  | Reverse: | TGTAAGAGGCAGGAGGCGTGAC |
| *SPATA19* | Forward: | AGATGGAGCCATACCCGTGTCTTC |
|  | Reverse: | TGGGAAATGCTTCGTCTCACTTGC |
| *TNP1* | Forward: | TCAAGAGAGGCGGCAGCAAAAG |
|  | Reverse: | GATCACAAGTGGGAGCGGTAACTG |
| *AKAP3* | Forward: | TTGTGTCTGCCCTGCTTCTGATTC |
|  | Reverse: | GGGTCTTGGTGATGGGAAAGTTGG |
| *AKAP4* | Forward: | AGCCCTTCCACCGACTCACTG |
|  | Reverse: | CCTTCGCTTGCTGGGTTAGATGG |
| *SPATA5* | Forward: | ATGGCATTGGCTCAGAAGGAAGTG |
|  | Reverse: | TCGTCCAGGTCTTCGGAGTGC |
| *ROPN1* | Forward: | AGCAGTTTACCAAAGCCGCCATC |
|  | Reverse: | AACTCGCTCAGACCGCTCTCC |
| *ROPN1L* | Forward: | GGCAACCCAGAAAGTAGACACAGG |
|  | Reverse: | TCTCCATTGGCAGGCACAGATTTC |
| *ODF1* | Forward: | TGTGTGACCTCTACCCGTGCTG |
|  | Reverse: | CGATGGCTCTGATGGCTTTCCTC |
| *ODF2* | Forward: | GGAACCAGCATAAGGCGGAAGTAG |
|  | Reverse: | TCGGCATACTCCTCACTCTTCTCG |
| *TCP11* | Forward: | ATCTTGCTGTCACTGCTGTTACCG |
|  | Reverse: | CGTGTTCTGCCTCCTGCTTGATC |
